# Understanding silicone elastomer curing and adhesion for stronger soft devices

**DOI:** 10.1126/sciadv.adv2681

**Published:** 2025-07-16

**Authors:** Te Faye Yap, Jasmine Klinkao, Sofia Urbina, Neethu T. Pottackal, Marquise D. Bell, Anoop Rajappan, Denizhan Yavas, Daniel J. Preston

**Affiliations:** ^1^ Department of Mechanical Engineering, Rice University, Houston, TX 77005, USA.; ^2^ Department of Mechanical Engineering, University of Hawaii at Manoa, Honolulu, HI 96822, USA.; ^3^ Department of Materials Science and Nanoengineering, Rice University, Houston, TX 77005, USA.; ^4^ Department of Physics and Engineering Physics, Tulane University, New Orleans, LA 70118, USA.; ^5^ Rice Advanced Materials Institute, Rice University, Houston, TX 77005, USA.

## Abstract

Silicone elastomers are widely used in biomedical devices and soft machines because of their compliance, inertness, and biocompatibility. Their sol-gel transition during curing enables mold casting and layer-by-layer manufacturing, allowing the fabrication of fully elastomeric and hybrid soft-rigid devices. However, controlling adhesion at material interfaces remains elusive, especially under diverse temperature conditions. This study introduces a framework that relates adhesion strength to a dimensionless reaction coordinate coupling time and temperature. This reaction coordinate can be used to predict the transition from bulk fracture to adhesive failure, which is crucial to create robust devices with strong interfaces. Using this framework, we fabricated elastomeric robotic actuators and demonstrated 3D printing with direct ink writing. The actuators achieved 50% higher curvature with the same design, and the 3D-printed parts exhibited over 200% improvement in interlayer adhesion. This work serves as a tool for optimizing interfacial adhesion for soft materials across different fabrication approaches.

## Teaser

The cure extent of silicone elastomers informs adhesion strength, enabling durable soft devices with robust interfaces.

## INTRODUCTION

A unique combination of desirable material properties, including inherent compliance, biocompatibility, moldability, and chemical stability, has made silicone elastomers highly versatile materials used across a wide range of sectors. Applications of silicone elastomers include food-grade molds and cookware owing to their thermal stability (*1*), antifouling and water-repellant coatings benefiting from their low surface energy (*2*, *3*), and gaskets for sealing and vibration dampeners leveraging their compliant nature (*4*, *5*). In addition, the biocompatibility of silicone elastomers has led to widespread use in medical devices such as prostheses (*6*), catheters (*7*), bioadhesives (*8*, *9*), and biointegrated electronics that combine functionality with compatibility for long-term use on, or in, the human body (*10*, *11*). Despite their broad use, one of the main outstanding challenges across these fields is achieving a strong, reliable adhesion at interfaces, especially for devices that are assembled from multiple components. When bonding layers, poor interfacial adhesion can lead to delamination, premature mechanical failure, and reduced performance (*12*–*14*).

The field of robotics has also greatly benefited from the integration of silicone elastomers, particularly in the development of the burgeoning field of soft robotics. Fully soft robots, constructed entirely from compliant materials like silicone elastomers, are capable of effectively navigating unstructured terrain, interacting safely with diverse environments, and handling delicate specimens in a nondestructive manner. The ability to interact with their surroundings without causing damage often enables soft robots to outperform traditional rigid robots (*15*–*18*). Beyond fully soft robots, increasingly, hybrid rigid-soft robots are also being explored to achieve a broader range of functionalities. These hybrid designs are often inspired by the musculoskeletal systems of natural organisms, which capitalize on rigid load-bearing structures for control and precision, while soft, compliant materials enable adaptability, safe interaction, and added degrees of freedom (*19*, *20*).

**Fig. 1.**
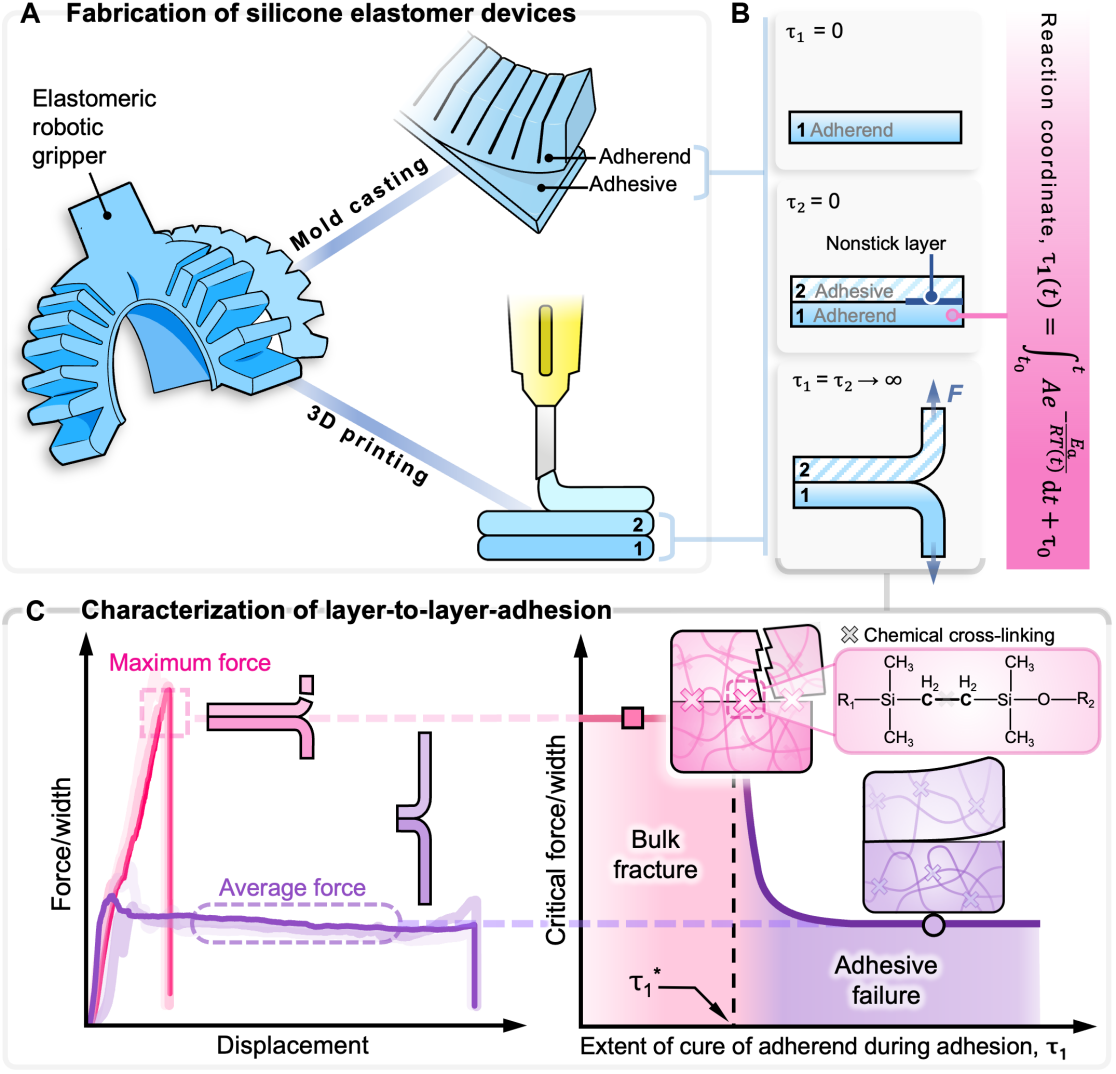
Studying the layer-to-layer adhesion of silicone elastomers as a function of cure extent. (**A**) Silicone elastomeric devices are often fabricated via mold casting or 3D printing. In these approaches, the material used to construct the components of a soft device also functions as the adhesive—in its liquid prepolymer state—to assemble components in mold casting or to adhere to preceding layers during layer-by-layer DIW. (**B**) We study adhesion strength at the interface between an adherend (layer 1) and adhesive (layer 2) by quantifying the critical peel force as a function of reaction coordinate (cure extent) of the adherend when the adhesive is applied, and we perform T-peel tests after both layers are fully cured (τ_1_, τ_2_→∞). (**C**) Sample data showing force-per-width results obtained from T-peel tests. Samples that experience bulk fracture reach a maximum force before bulk fracture, while samples that fail adhesively peel apart at the interface with the critical peel force required to delaminate the interface defined by the plateau during steady-state peeling. The maximum force and average steady-state peeling force are used to quantify bulk and adhesive failure, respectively. A critical τ_1_^*^ exists where the mode of failure transitions from bulk fracture to adhesive.

The fabrication of soft robots with unique geometries is enabled by the processability of silicone elastomers. Two-part platinum-catalyzed silicone elastomers undergo a sol-gel transition during curing, transforming from a liquid-like prepolymer to an elastic solid. This phase transition is advantageous for mold casting, a technique widely used to create both fully soft and hybrid robots, as well as other elastomeric devices (*18*, *21*–*23*). In this process, individual components are fabricated by casting liquid prepolymer into a mold, where the prepolymer progressively cures into an elastic solid over time. Once cured, these components can be demolded and further assembled into devices (Fig. 1A). For fully elastomeric devices, the same silicone material in its liquid-like prepolymer state can serve as an adhesive to bond layers or components together during assembly. However, ensuring strong interfacial adhesion during assembly remains a challenge, particularly for devices like pneumatic actuators that experience high pressure and strain, where delamination often leads to premature failure (*24*–*28*). For instance, prior work by Yirmibesoglu *et al.* (*28*) compared the performance of 3D-printed and molded pneumatic network (pneu-net) actuators. Their study found that the molded pneu-nets, which required demolding and subsequent assembly, failed at a lower actuation pressure compared to their 3D-printed counterparts. The authors attributed the better performance of the 3D-printed pneu-nets to the “sewing thread effect,” similar to that of fiber-reinforced actuators; as we show in this work, the diminished performance of the molded actuators is instead likely due to weak adhesion at the bonded interface, resulting from overcuring before assembly.

An emerging technology in soft-device fabrication that has gained traction in recent years is additive manufacturing (or 3D printing) of thermosetting elastomers, including platinum-catalyzed silicone elastomers (Fig. 1A). Compared to mold casting, 3D printing offers additional design flexibility, enabling rapid prototyping of complex, multilayered structures that would be challenging to achieve with traditional mold-casting methods. The ability to 3D print with unmodified silicone elastomers is particularly attractive because these materials offer a wide range of desirable mechanical properties, such as extreme softness and the ability to withstand high strains. These properties are not typically exhibited by thermoplastic-based parts fabricated using traditional additive manufacturing techniques, such as fused filament fabrication. Researchers and industry have increasingly focused on formulating materials (e.g., silicone resins from Formlabs) and optimizing additive manufacturing techniques for commercially available silicones to enable compatibility with direct ink writing (DIW) and vat polymerization (*24*, *25*, *28*–*30*). However, as with mold casting, weak adhesion between printed layers remains a critical limitation, leading to diminished mechanical properties and precluding the final printed part from fully realizing the bulk material’s desirable inherent properties. Prior studies have identified poor interlayer adhesion as a potential reason why printed silicone structures fail to attain the expected mechanical performance (*29*–*31*). This limitation highlights the importance of improving our understanding of the layer-to-layer adhesion to optimize processing parameters and design materials that promote robust interfacial adhesion and maximize the mechanical benefits that silicone elastomers can provide.

Different methods have been proposed to circumvent issues related to delamination in elastomeric devices. Plasma treatment has been shown to improve bond strength between polydimethylsiloxane (PDMS) components (*13*, *32*–*35*); however, this treatment method is not effective on all commercially available platinum-catalyzed silicone elastomers because of potential migration of silicone oils to the surface that interrupt plasma bonding (*36*). Alternative fabrication methods have been developed to create monolithic devices, such as using sacrificial or flexible cores that can be easily removed or leveraging fluid mechanics and interfacial flows to create programmable voids, thereby circumventing the need for bonded joints altogether (*26*, *37*–*39*). These approaches are effective for enhancing the mechanical performance and strength of the device through innovative geometries and actuation designs. However, in complex scenarios where multiple parts are required to assemble a device or additive manufacturing, there is still a need to develop a solution to enable strong layer-to-layer adhesion. One way to improve the adhesion between layers is to consider the extent of cure of the elastomer during bonding. Prior studies have attributed overcuring of components (i.e., when the silicone elastomer has approached complete cross-linking) before bonding as a potential reason for weak interfacial adhesion (*29*, *40*, *41*). Therefore, the cure extent of the components or layers during bonding represents an important consideration for improving adhesion strength. 

Peel tests are often used to quantify the critical peel forces required to delaminate interfaces to ensure the integrity of bonding between components (*42*, *43*). Extensive work in the literature has focused on studying the adhesion between dissimilar materials (*42*–*45*). Commonly examined materials include pressure-sensitive adhesives and structural adhesives (such as epoxies and cyanoacrylates) that bond chemically to a substrate. The adhesion strength between the adhesives and substrate is tested. The adhesion between similar materials, for silicones and hydrogels, has been investigated under various bonding conditions, such as plasma activation, silane couplings, bridging polymer networks, and acrylate-based adhesives (*13*, *34*, *35*, *46*); however, there remains a need to further investigate how cure extent influences adhesion strength between similar material surfaces. A prior study by Walker *et al.* (*29*) investigated the effect of cure extent on the resulting failure modes of modified silicone elastomers by varying the time between application of successive layers at a specific constant temperature. Their work provided important insights into the relationship between cure extent and adhesion; however, it primarily focused on isothermal curing conditions. In practice, platinum-catalyzed silicone elastomers are often subjected to varying temperature profiles to control curing times, with elevated temperatures commonly used to accelerate fabrication (*23*, *47*–*49*). As a result, curing histories are not defined by a single, constant temperature. To enable broader application and reliable interfacial bonding across diverse curing conditions, a framework that captures the effects of arbitrary, time-varying temperature profiles is crucial to optimize the bond between adherend and adhesive and ensure the mechanical durability of the silicone-based devices.

In prior work, we developed a modeling framework that describes the cure extent with a nondimensional reaction coordinate, which is a function of both time and temperature (*50*). The reaction coordinate was determined on the basis of the superposition of dynamic moduli—experimentally measured using small-amplitude oscillatory shear tests at various temperatures—which was normalized by a characteristic timescale, the gelation time. The gelation time was determined as the time at which the storage modulus **G*′* exceeds the loss modulus **G*″*. Upon nondimensionalizing the dynamic moduli with the gelation time, the curing reaction was shown to exhibit self-similarity, and the nondimensional timescale therefore acts as a reaction coordinate that informs us of the extent of curing. By virtue of how the time variable was normalized, the gelation point corresponds to a τ value of 1. A modeling framework based on the Arrhenius equation coupled with data from thermorheological experiments allows us to determine the cure extent for arbitrary heating conditions (Eq. 1)

$$\tau \left(t\right)=\int_{t_{0}}^{t}Ae^{-\frac{E_{a}}{\mathrm{RT}\left(t\right)}}\;dt+\tau_{0}$$ (1)

The integral on the right-hand side of Eq. 1 can be computed numerically using a fourth-order Runge-Kutta (Dormand-Prince) method, where τ_0_ = 0 at *t* = 0. The variable *R* is the universal gas constant, *E*_a_ is the activation energy, *A* is the frequency factor, and τ_0_ is the initial reaction coordinate. Activation energy and frequency factor values for the silicone elastomers studied in this work (i.e., Ecoflex 00-30 and Dragon Skin 30) were obtained by referring to our prior work (fig. S1), which focused only on curing and not on adhesion.

In this work, we leverage our understanding of the nondimensional reaction coordinate τ to systematically characterize the adhesion strength between the adherend and adhesive as a function of both the curing time and temperature of commercially available platinum-catalyzed silicone elastomers without chemically modifying the material. To this end, we quantified the adhesion strength as a function of the reaction coordinate of the adherend during the application of the adhesive τ_1_ by performing T-peel tests (Fig. 1). Results from our experiments reveal that past a certain reaction coordinate, τ_1_, the mode of failure transitions from bulk fracture to interfacial delamination, validating the hypothesis that overcuring leads to weak adhesion at the bonded interface. In addition, we investigated the effect of sample thickness on the forces experienced during the different modes of failure. We used finite element modeling to rationalize the experimental results and provide insight into the different failure modes by studying the stress profiles and adhesion energies during peel tests. These findings allow us to use this modeling framework as a guideline to improve, or to selectively program, the adhesion strength of elastomeric devices without the need for additional treatments that could alter the inherent material composition, thus broadening the design space for the manufacturing of silicone elastomer devices. As a demonstration of the applicability of this framework for adhesion strength as a function of the extent of curing, we fabricated molded pneu-net soft robotic actuators, where two separate components were adhered at varying τ_1_ values. We quantified the maximum curvature and pressure attained before failure, as well as the physical location of failure; here, avoiding adhesive failure on the basis of our modeling framework enabled 50% greater pneu-net curvatures before device failure. Expanding the framework to additive manufacturing, we used DIW to 3D print T-peel geometries while varying the time (i.e., reaction coordinate) between printing subsequent layers, indicating that careful control of printing parameters guided by our modeling framework can increase interlayer adhesion by over 200%, thereby improving 3D printing capabilities for silicone elastomers.

## RESULTS

### Quantifying the adhesion strength between adherend and adhesive

During the fabrication of a fully elastomeric device, the liquid prepolymer often acts as an adhesive between components. To study the adhesion strength, we focused on the interface between the component (which we designate as the adherend or “layer 1”) and the adhesive (“layer 2”; Fig. 1A). We generated a series of samples for which we varied the reaction coordinate of the adherend, τ_1_, when the adhesive is applied, and we performed T-peel tests to quantify the relevant forces during failure. To fabricate the samples (fig. S4), we dispensed the mixed prepolymer (Materials and Methods) to fill half of the 3D-printed, rectangular mold (fig. S2) to create the adherend (or the first layer), which has a thickness *t* of 2.5 mm (with additional thicknesses characterized in the following sections). To quantify a baseline for the failure behavior, we fabricated samples and allowed these to cure at room temperature. To compute the instantaneous reaction coordinate of the adherend as it is curing, we recorded the real-time temperature of the adherend using a thermocouple connected to a computer-based data acquisition device to account for any slight temperature variations in room temperature. Simultaneously, we computed the instantaneous reaction coordinate values using a custom MATLAB script that incorporates the modeling framework (Eq. 1) and the temperature (Fig. 1B). A thin nonstick layer (40 mm by 25 mm by 0.1 mm) is placed over one end to define the peel arms of the sample (to be secured by the grips on the universal testing machine during T-peel testing). The adhesive, represented by a batch of freshly mixed prepolymer (τ_2_ ≈ 0), was prepared in the same manner and applied onto the adherend. The τ_1_ value during the application of the adhesive to the adherend was recorded for each specific sample. Each sample was then tested after both layers had been allowed to fully cure, where τ_1_ and τ_2_→∞ correspond to values much greater than (at least 2×) the curing duration indicated by the manufacturer’s data sheet (which was τ > 4 for Ecoflex 00-30 and τ > 15 for Dragon Skin 30).

**Fig. 2.**
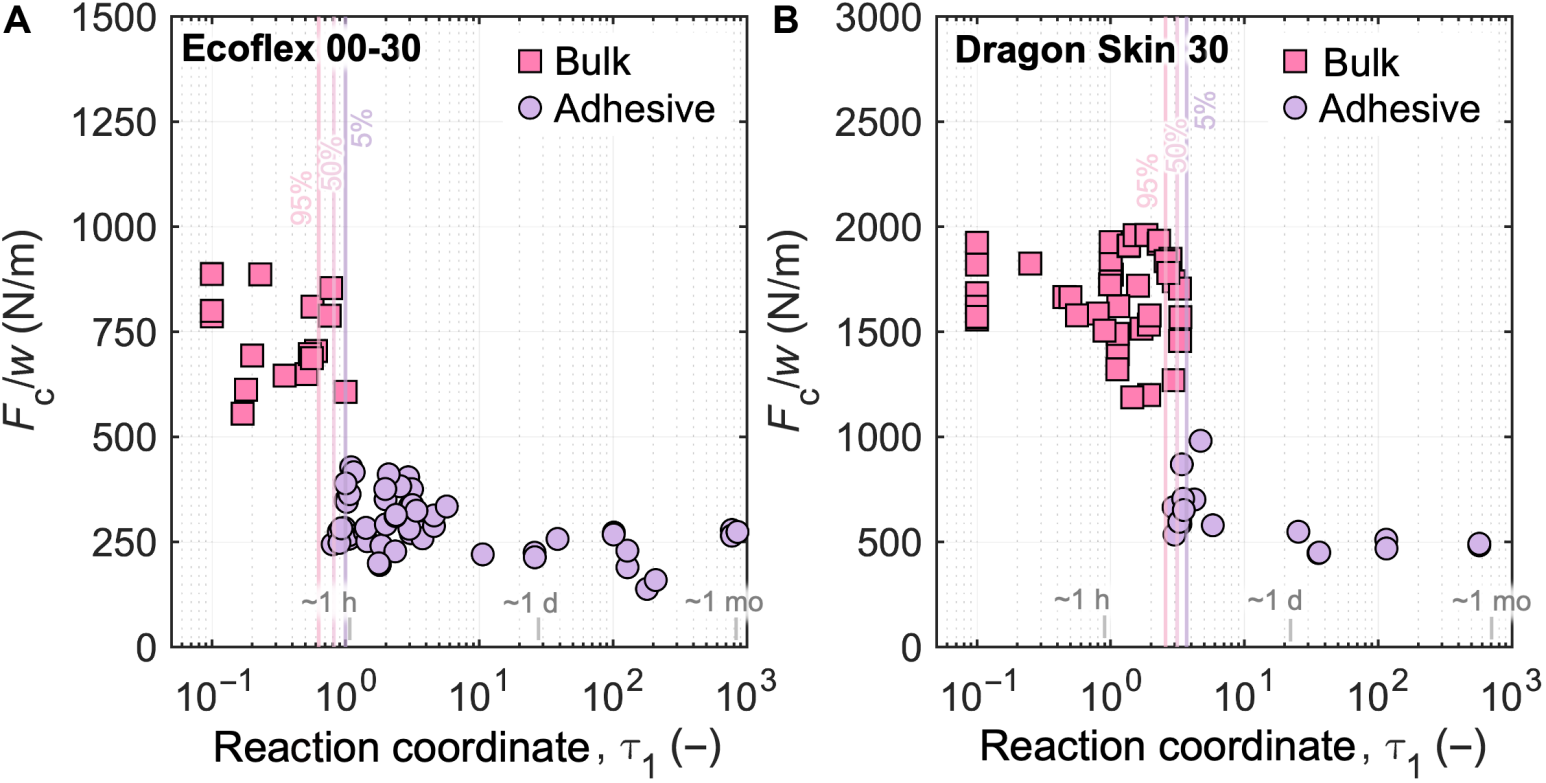
T-peel test results as a function of reaction coordinate. Critical peel force values *F*_c_ for (**A**) Ecoflex 00-30 and (**B**) Dragon Skin 30 were plotted against τ_1_ for each independently fabricated sample. A timescale is included for each elastomer to provide the duration of curing corresponding to the reaction coordinate values at room temperature (23°C). The 95, 50, and 5% probability of bulk fracture occurring are highlighted in the plots. The τ_1_ value corresponding to 50% probability is designated as the transition reaction coordinate τ_1_^*^, which are 0.82 and 3.13 (table S2) for Ecoflex 00-30 and Dragon Skin 30, respectively. The average thicknesses of the samples determined from the peel arms are *t* = 2.46 mm (SD = 0.19, *n* = 71) for Ecoflex 00-30 and *t* = 2.43 mm (SD = 0.16, *n* = 54) for Dragon Skin 30.

We fabricated samples with varying τ_1_ values and performed T-peel tests using a universal testing machine (Materials and Methods). The samples were subjected to a quasi-static strain rate of 100 mm/min, and the critical peel force values were normalized by the width as *F*_c_/*w*. We categorized the modes of failure as bulk fracture and adhesive failure (Fig. 1C). Bulk fracture occurs because of failure within the material itself, for which we quantified the critical peel force *F*_c_ that resulted in failure using the maximum force per width at break; conversely, adhesive failure occurs because of fracture at the bonded interface between the layers, which results in delamination, and we characterized this mode of failure with the average plateau force per width during steady-state peeling, where we took an average of 80% of the data within the steady-state peeling region (fig. S6) (*45*). We hypothesized that samples with low τ_1_ values (reaction coordinate of the adherend during the application of adhesive) would experience bulk fracture; as a limiting case, an arbitrary line bisecting a bulk freshly mixed reservoir of uncured prepolymer would correspond to τ_1_ = 0, where the sample behaves as a homogeneous material with no interface between the two layers. As τ_1_ increases, the failure mode transitions from bulk to adhesive, where a discernible interface exists and the two layers can be peeled apart at this interface (Fig. 1C). Figure 2 (A and B) shows results from the T-peel test plotted as a function of τ_1_ for Ecoflex 00-30 and Dragon Skin 30, respectively. Each data point on the plot represents an individual T-peel test on an independently fabricated sample. The pink squares represent samples that experienced bulk fracture, and the purple circles represent samples that failed adhesively (determined by observation and from the force versus displacement data).

The experimental results corroborate our hypothesis: A transition from bulk fracture to adhesive failure occurs at a critical reaction coordinate τ_1_^*^, where τ_1_^*^ ≈ 0.8 and 3.0 (exact values tabulated in table S2) for Ecoflex 00-30 and Dragon Skin 30, respectively. We determined τ_1_^*^ using logistic regression based on our experimental data to determine the 50% probability of bulk fracture and adhesive failure. In addition, we also indicate the 95% and 5% probability for bulk fracture in Fig. 2, table S2, and fig. S7. We attribute the transition in failure modes to the high density of covalent bonding across the interface when the adhesive is applied to the adherend at low τ_1_, allowing the sample to behave more like a monolithic component; meanwhile, at higher τ_1_, reduced or negligible covalent bonding occurs between the layers, with only physical (e.g., van der Waals) bonding holding distinct layers together, resulting in relatively low adhesion energy between the layers (*51*, *52*). The scatter in the experimental data could be attributed to several factors, including slight variations in thickness, mixing ratios, grip alignment during testing, premixing quality of individual part A and part B components, surface imperfections, potential batch-to-batch variation in elastomer formulations, and environmental conditions (*53*, *54*). A timescale is included in Fig. 2A to provide an intuition of the curing duration corresponding to the reaction coordinate values at room temperature (23° ± 1°C). The timescale reveals that curing Ecoflex 00-30 for more than 1 hour at room temperature would lead to decreased adhesion between the adherend and adhesive, posing a high risk of delamination—for instance, if cast Ecoflex 00-30 parts were left in their molds for more than an hour at room temperature before “gluing” them together with fresh prepolymer, adhesive failure would likely occur. These findings suggest that we can anticipate and predict the failure modes of devices fabricated by molding and casting or 3D printing by quantifying the reaction coordinate of the adherend during adhesion for different commercially available silicone elastomers, providing fabrication guidelines to ensure strong bonding between components or layers without fundamentally modifying the material itself.

### Studying the effect of sample thickness on adhesion strength

**Fig. 3.**
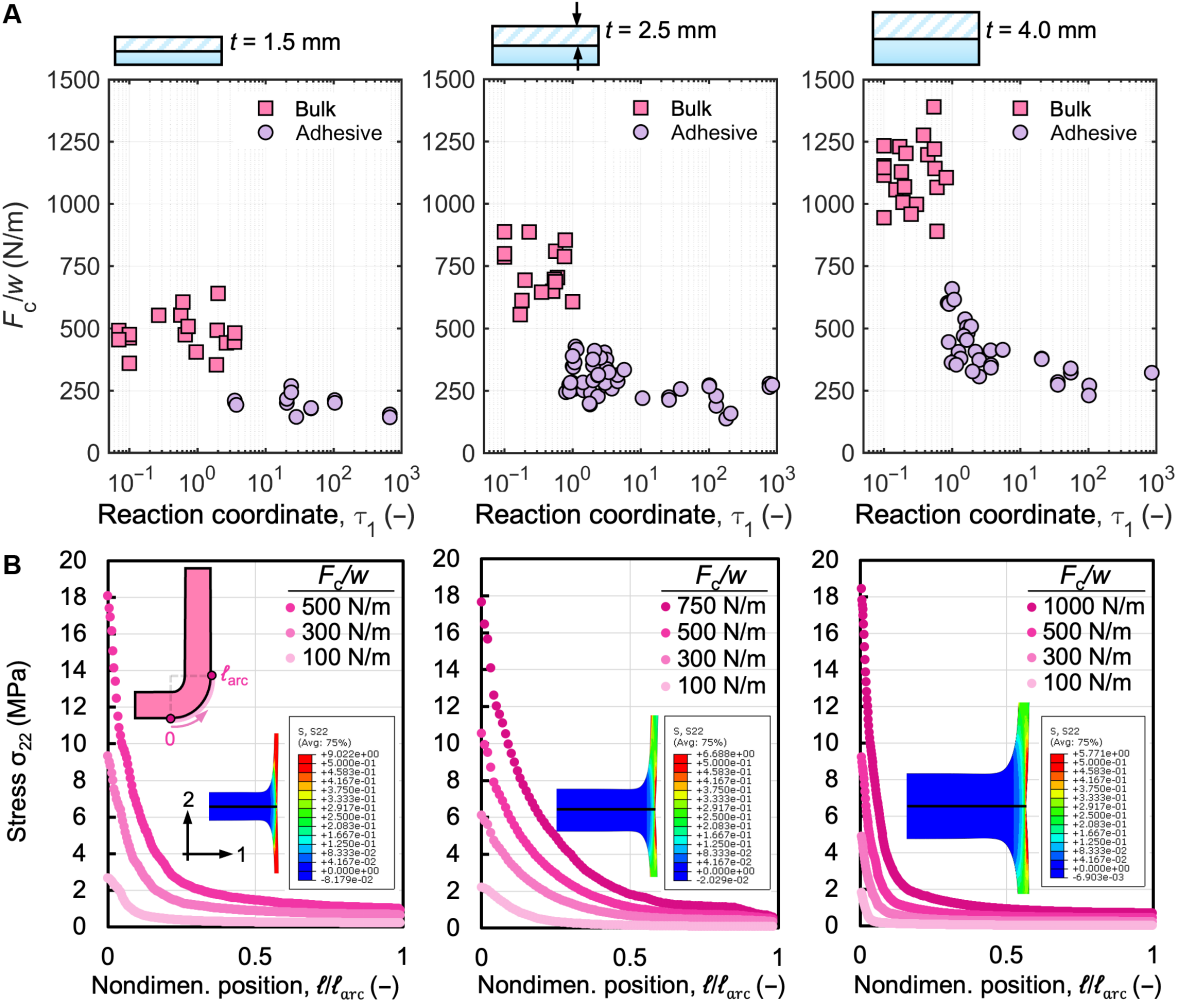
Analysis of the effect of sample thicknesses on T-peel test results. The critical peel force for failure was determined for Ecoflex 00-30 samples with a target thickness of (**A**) 1.5 mm (average = 1.55 mm, SD = 0.15, *n* = 32), 2.5 mm (average = 2.46 mm, SD = 0.19, *n* = 71), and 4.0 mm (average = 3.81, SD = 0.19, *n* = 56). (**B**) FEM results showing that bulk fracture occurs when the true stress at the interface achieves the true tensile strength of the material for all three thicknesses. Insets show the FEM simulation illustrating the magnitude of stress concentrations across three thicknesses when subjected to the same forces.

To study the effect of sample thickness on the adhesion strength and failure mode, we performed T-peel experiments on samples of two additional thicknesses, which were thinner (1.5 mm) and thicker (4.0 mm) than the 2.5-mm thickness previously tested with Ecoflex 00-30. The results in Fig. 3A show that the critical peel force per width for bulk fracture increases proportionally to the sample thickness. Given that failure occurs within the peel arm for samples that experience bulk fracture, the cross-sectional area (*w* × *t*) of the peel arms likely influences the maximum forces observed. To further understand the critical peel force values during bulk fracture, we used the finite element method (FEM) to investigate the stress profile of the samples during T-peel testing. Because of stress concentrations arising from the geometry of the T-peel sample, it is important to account for the true stresses experienced within the sample during the peel test. From the finite element analysis, bulk fracture occurs when the true stress in the peel arms approaches the true tensile strength of the material (fig. S8). Figure 3B presents the vertical stress component (σ_22_) within the material as a function of nondimensional distance from the bonding interface under different simulated peel forces for the three thicknesses studied. The inset highlights the vertical stress fields of each sample thickness experiencing the same peel force of about 300 N/m. The thinner samples exhibit higher stress near the interface within the arm as compared to the thicker samples. Using the experimentally determined approximate critical peel force for bulk fracture for each thickness, finite element analysis demonstrates that all three thicknesses experience similar stress values near the bonding interface, with σ_22_ ≈ 18 MPa during failure, which closely resembles the true tensile strength of the material (see the Supplementary Materials).

Analyzing the failure mode transition in Fig. 3A, the thickness of the sample plays a secondary role in influencing the value of the reaction coordinate τ_1_^*^, at which the transition from bulk fracture to adhesive failure occurs. For samples with thicknesses of 2.5 and 4 mm, τ_1_^*^ consistently occurs around 0.8. However, for the thinnest sample (1.5 mm), τ_1_^*^ is delayed, occurring at a higher value of approximately 3.5 (table S2). Early discussions in the literature on pressure-sensitive adhesive tapes with inextensible backings have provided some explanation regarding the effect of adhesive thickness on failure mode transitions (*43*); for samples that have weak adhesion at the interface, the material will still tend to undergo bulk fracture if the critical peel force required to fracture the material is lower than the force required to delaminate the interface. The material will continue to experience bulk fracture until the critical peel force required to delaminate the interface becomes lower than the critical peel force for bulk fracture, at which point it makes the transition to adhesive failure. In accordance with this line of inquiry, in the case of the sample with a thickness of 1.5 mm, the critical peel force required to induce bulk fracture at τ_1_ = 0.8 is likely lower than the critical peel force required for delamination, delaying the failure mode transition. The delayed transition occurs because the critical delamination force varies near τ_1_^*^ and decreases with increasing τ_1_, eventually plateauing. As the value of τ_1_ increases, the force required to fracture the bulk material exceeds the critical delamination force near τ_1_^*^ = 3.5, resulting in adhesive failure.

**Fig. 4.**
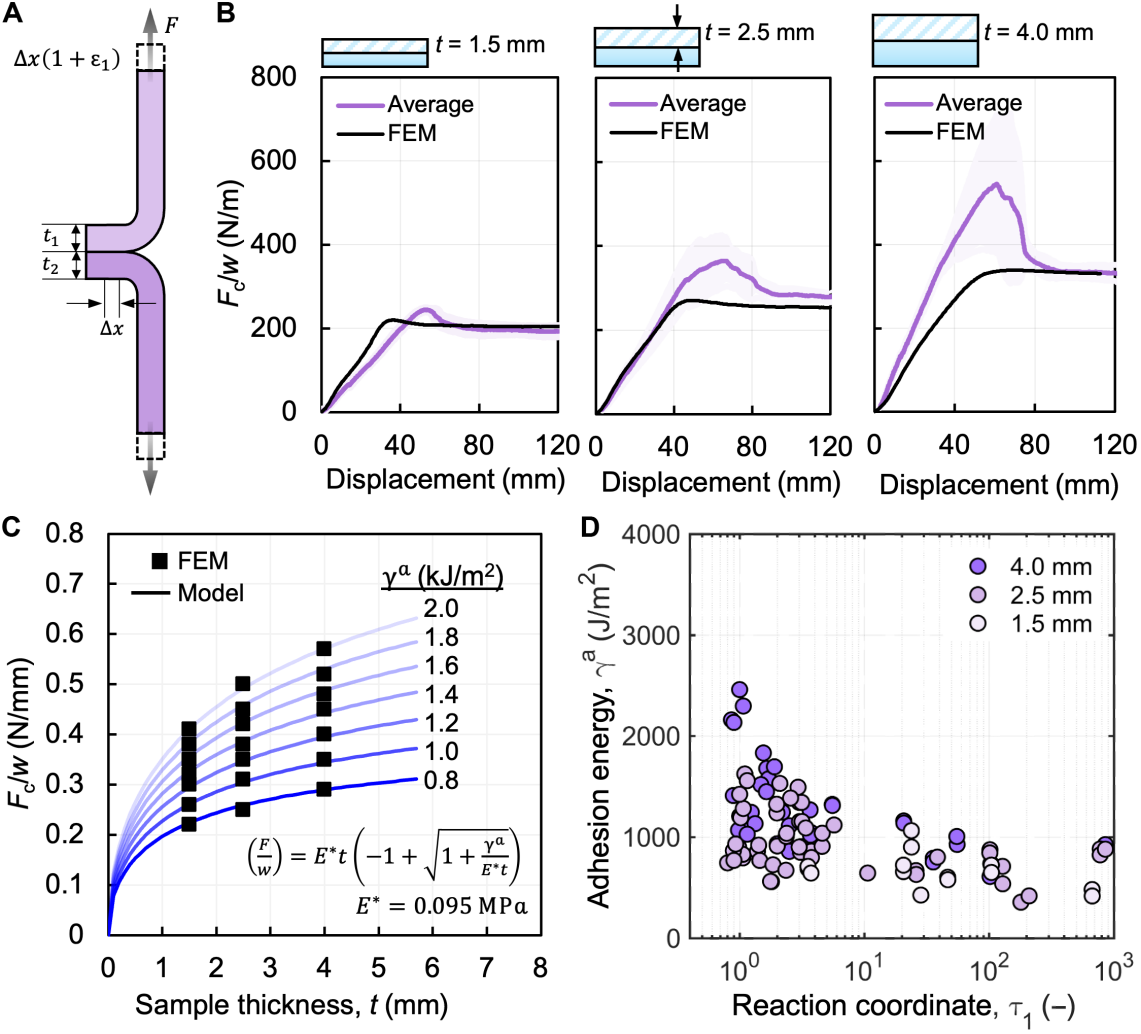
Effect of thickness on the adhesion energy. (**A**) Schematic depicting a T-peel sample undergoing delamination with relevant variables. (**B**) The finite element model was calibrated on the basis of the data averaged across three samples made of Ecoflex 00-30. The shaded region represents the 95% confidence interval. (**C**) Using the calibrated FEM, the effective modulus E^*^ was determined by fitting a simplified Eq. 6 (assuming *t*_1_ = *t*_2_ = *t*) to FEM simulated force values at 1.5, 2.5, and 4.0 mm. (**D**) Adhesion energy determined using E^*^ in Eq. 6 and experimentally determined force values are shown for all three thicknesses studied in this work, indicating similar values across all thicknesses.

To investigate the effect of thickness during the adhesive failure mode, we can determine the adhesion or fracture energy for elastic peel arms using Griffith’s approach based on linear elastic fracture mechanics (*55*–*57*). The total energy in the system consists of the work done at a constant force to strain the interface (*W*), the strain energy stored in the stretched peel arm (*U*_e_), and the interfacial adhesion energy (γ^a^). Figure 4A depicts a T-peel sample undergoing interfacial delamination, along with the relevant parameters in the system. By conservation of energy, γ^a^ is defined by

$$\gamma^{a}=\frac{\partial W}{\partial A}-\frac{\partial U_{e}}{\partial A}\;$$ (2)

where *A* represents the unit crack growth area due to delamination and can be calculated as *w*∆*x*. The work done by the critical peel force during adhesive failure and creation of newly formed surface over a distance of ∆*x* can be expressed by accounting for the strain ε in both peel arms as

$$W=F_{c}∆x\left[\left(1+\varepsilon_{1}\right)+\left(1+\varepsilon_{2}\right)\right]$$ (3)

The delamination process increases the elastic strain energy *U*_e_ as more exposed area is generated. We calculated the elastic strain energy by summing the energies stored in both arms of the T-peel sample, which is expressed as

$$U_{e}=w∆x\left(t_{1}\int_{0}^{\varepsilon_{1}}\sigma d\varepsilon +t_{2}\int_{0}^{\varepsilon_{2}}\mathrm{\sigma d\varepsilon}\right)$$ (4)

and by solving the equation and substituting ε=σ/E and $\sigma =F_{c}\;/\;w\text{∆}x$

$$U_{e}=\frac{F_{c}^{2}∆x}{2\text{wE}}\left(\frac{1}{t_{1}}+\frac{1}{t_{2}}\right)$$ (5)

where σ is the stress and *E* is the modulus of elasticity of the silicone elastomer. Last, we determine the interfacial adhesion energy by substituting Eqs. 3 and 5 into Eq. 2, arriving at

$$\gamma^{a}=2\left(\frac{F_{c}}{w}\right)+\frac{1}{2E}{\left(\frac{F_{c}}{w}\right)}^{2}\left(\frac{1}{t_{1}}+\frac{1}{t_{2}}\right)$$ (6)

The equation can be further simplified for symmetric peel arm thicknesses, in which case *t = t*_1_ = *t*_2_. However, because of small deviations in the thickness of our samples, we decided to continue our calculations of γ^a^ using Eq. 6 with experimentally measured thickness values. Equation 6 assumes that the material is linear elastic, with a constant modulus *E*; however, the silicone elastomers display a moderate degree of nonlinearity in the adhesion energies examined. Therefore, to account for the nonlinear behavior of the silicone elastomer undergoing deformation, an effective modulus E^*^ is introduced in place of *E* (Fig. 4C) in Eq. 6, which we determined using finite element modeling (see the Supplementary Materials).

Figure 4B shows the comparison of FEM simulation results and experimental data averaged across three experiments (fig. S9). We calculated the adhesion energy using the modified Eq. 6 and experimental force values for all samples that experience adhesive failure from Fig. 3A. We observed that the adhesion energy is independent of sample thickness and displays an inverse relationship with the reaction coordinate τ_1_ when near τ_1_^*^ and eventually plateaus as the reaction coordinate increases (Fig. 4D). The adhesion energy values at the plateau region are approximately 1000 J/m^2^, which is similar to the γ^a^ of tough hydrogels (*8*, *44*), whereas the typical adhesion energy when interfacing two fully cured silicone elastomers is on the order of 0.1 J/m^2^ (*45*, *51*, *58*). This high γ^a^ value likely arises because of the application of the adhesive (second layer) in its prepolymer state, allowing for interdiffusion of unreacted molecules into the adherend (first layer), where these molecules then either start cross-linking or entangle with the already cross-linked polymer network in the adherend layer, resulting in a stronger interface (*51*).

### Validating the critical reaction coordinate for time-varying temperatures

**Fig. 5.**
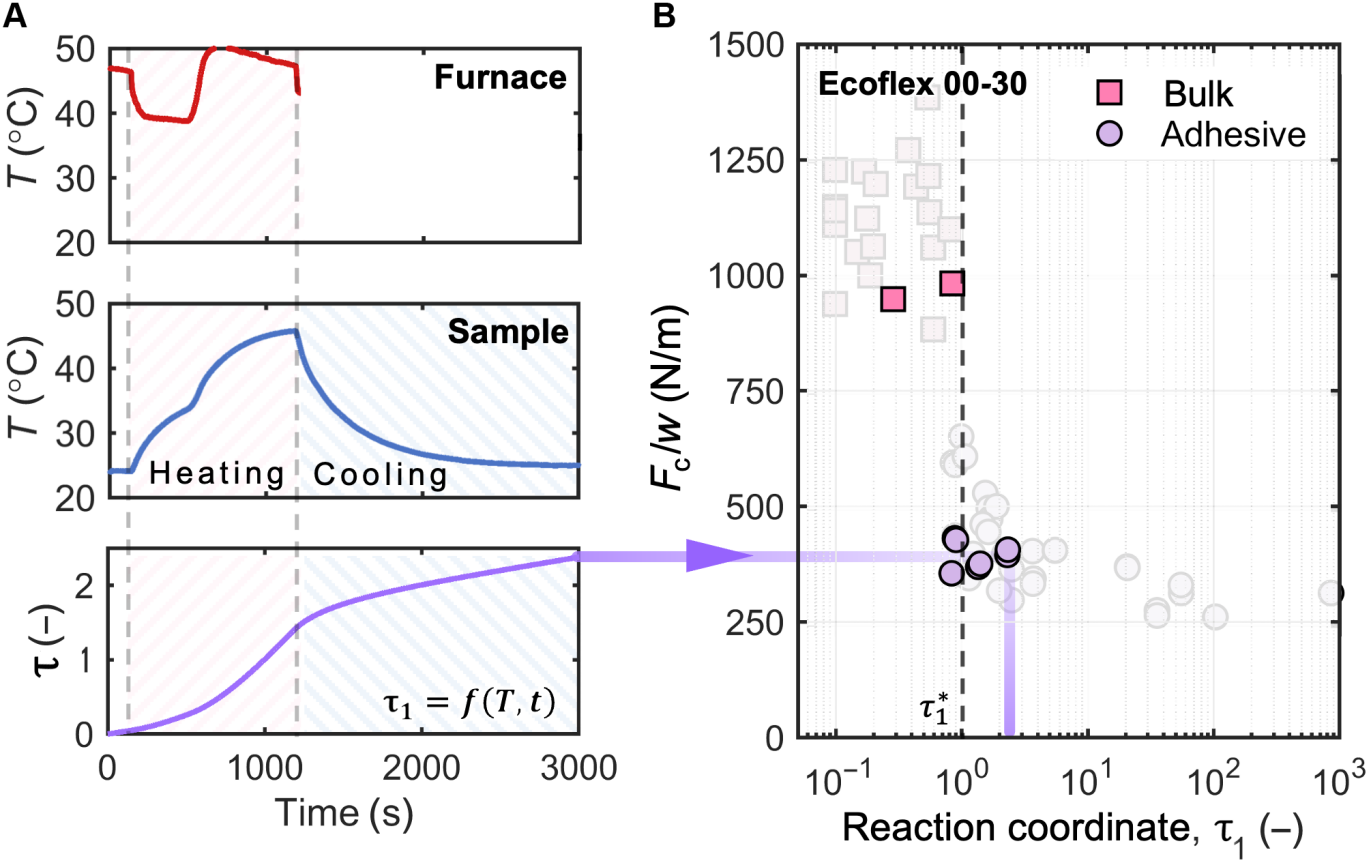
Verifying the effect of curing under time-varying temperature on failure mode based only on reaction coordinate. (**A**) One example of a real-time temperature profile of the furnace (top) and the sample that was heated in the furnace (middle). The bottom plot displays the calculated reaction coordinate as a function of time and temperature. The sample was placed in a preheated furnace at *t* = 130 s, then removed at *t* = 1200 s, and allowed to cool to room temperature. The second layer of prepolymer was applied at *t* = 3000 s, creating a T-peel sample with a τ_1_ of 2.5. (**B**) T-peel tests were performed on nine Ecoflex 00-30 samples that were all subjected to elevated temperature curing, and the resulting *F*_c_ was plotted against the predicted τ_1_ values. The results obtained for samples that were allowed to cure in the oven were overlaid on data from Fig. 3A, demonstrating the capability of using the reaction coordinate to consistently predict the mode of failure.

All the T-peel samples tested in the preceding sections were fabricated at constant room temperature, with only slight temperature variations (±1°C), to establish a baseline. In this section, we show the capabilities of using the reaction coordinate and our modeling framework to predict the mode of failure for samples that are cured at time-varying elevated temperatures. Studying the failure modes under these curing conditions is important because elevated temperatures are commonly used during the fabrication of soft devices to accelerate the curing reaction of platinum-catalyzed silicone elastomers. Samples were fabricated in a manner similar to the approach described above, where a thermocouple records the temperature of the interface of the first layer as it is curing, but, here, the sample is cured in a furnace where it is exposed to elevated temperatures. Figure 5A shows an example of a time-varying temperature profile of the furnace and the sample that is being cured. The furnace temperature fluctuates because of the bang-bang controller in place, resulting in overshooting and undershooting around the setpoint temperature. The first layer of the sample was allowed to partially cure in the furnace and then removed to cool to room temperature while curing continued. The bottom plot in Fig. 5A shows the reaction coordinate τ_1_ displayed as a function of sample temperature. The specific τ_1_ of the sample during the application of the adhesive layer was recorded for use in Fig. 5B. Figure 5B shows the *F*_c_ determined from T-peel tests for nine samples that were subjected to unique heating conditions and their corresponding τ_1_ values. We overlaid our experimental results on data shown in Fig. 3A, and the results indicate that we can consistently predict the mode of failure even for time-varying temperature conditions using the reaction coordinate. With this framework, strong adhesion can be achieved consistently and is not limited to specific curing conditions.

### Fabricating molded pneu-net actuators with varying adhesion strengths

**Fig. 6.**
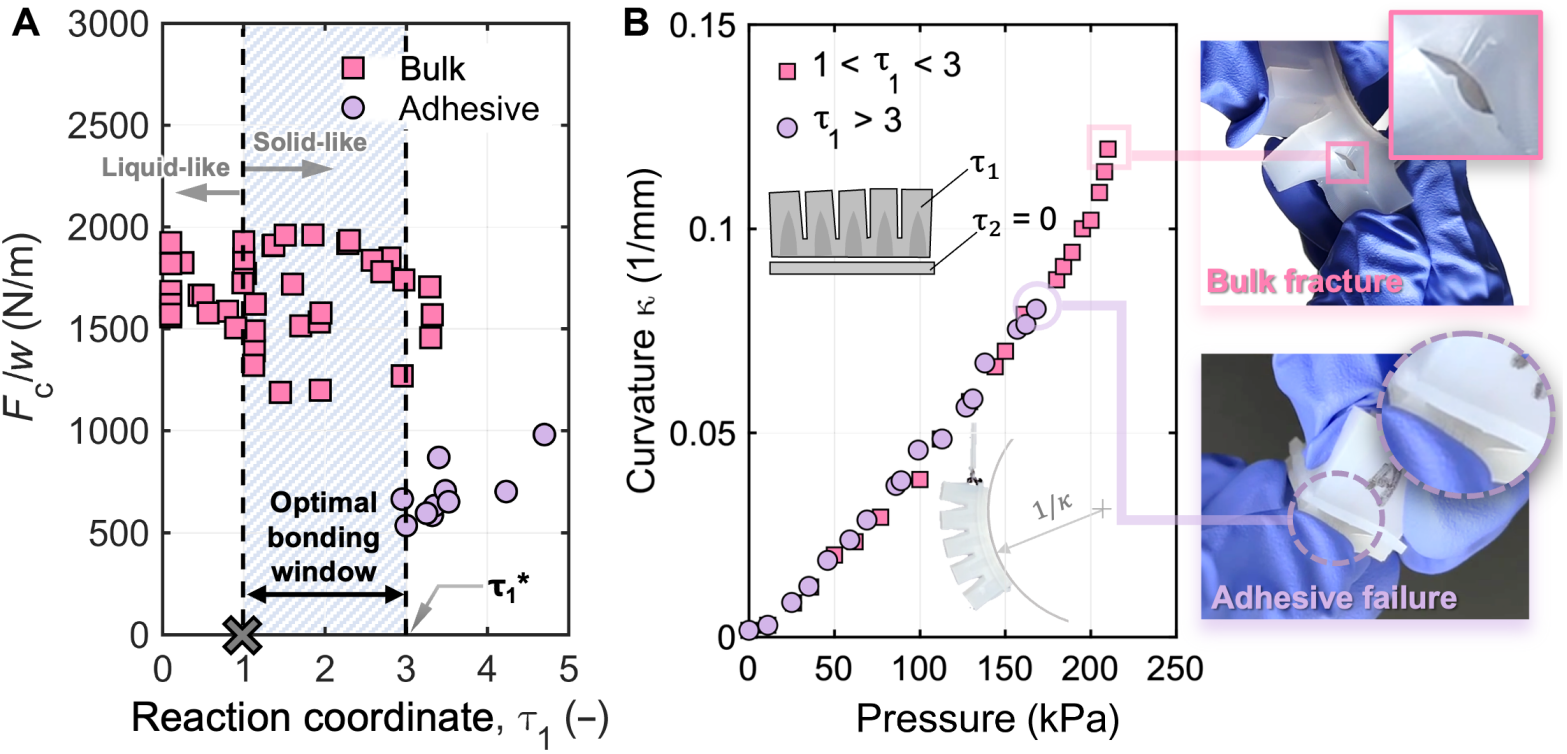
Fabricating pneu-net actuators with varying adhesion strengths. (**A**) The optimal bonding window (1 < τ_1_ < τ_1_^*^) for Dragon Skin 30 to achieve strong adhesion while exhibiting solid-like behavior to allow for demolding is highlighted in blue. (**B**) Pneu-nets were fabricated within the optimal bonding window or beyond τ_1_^*^ and were pressurized. The results show that the pneu-net fabricated within the optimal bonding window was able to withstand higher pressures and curvatures and failure occurred within the body of the actuator, while the pneu-net fabricated beyond τ_1_^*^ experienced delamination at the interface.

Revisiting the reaction coordinate, the nondimensional variable τ originated from normalizing the dynamic curing behavior with a characteristic timescale, which was chosen to be the gelation time (*50*). The gelation time represents the time at which a viscoelastic material transitions from a liquid-like to a solid-like state; therefore, τ = 1 marks this transformation. Leveraging this knowledge, elastomers that exhibit a τ_1_^*^ greater than 1 present a unique opportunity because they begin exhibiting solid-like behavior, which allows them to be demolded, while the reaction coordinate is still low enough for strong adhesion with a freshly mixed prepolymer adhesive. We observe that Dragon Skin 30 exemplifies this behavior with a τ_1_^*^ of approximately 3. Figure 6A highlights the region where Dragon Skin 30 starts becoming more solid-like and could be demolded and bonded, offering an optimal bonding window for achieving strong interfacial adhesion between 1 < τ < 3 in this case. To validate the utility of our framework, we applied it to the fabrication of a pneu-net actuator, a common soft robotic component. A pneu-net actuator typically consists of two main components: the main body, which encloses chambers that expand when pressurized, and an inextensible base that resists expansion. These components are commonly cast separately, demolded, and assembled by bonding with liquid prepolymer as an adhesive (fig. S16).

We fabricated two pneu-net actuators, curing the main body to either a τ_1_ within the optimal bonding window (between 1 and τ_1_^*^) or beyond τ_1_^*^. Once the targeted τ_1_ was reached, the main body was demolded and adhered to the base strain-limiting layer—a composite of an inextensible fabric (Supplementary Text and figs. S10 and S11) and freshly mixed prepolymer (τ_2_ = 0). This process of fabrication mimics the T-peel samples discussed earlier, where the adherend was cured to a specific τ_1_, and the adhesive was applied; in some cases, however, both the body and base layers are considered adherends and are joined by the adhesive prepolymer (fig. S16). We evaluated the integrity of the resulting bond strength by pressurizing the pneu-net actuators at a constant flow rate of 200 ml/min, recording the applied pressure (fig. S12), and observing the failure mode. We then determined the curvature of the pneu-net by postprocessing the recorded videos of the experiment. The curvature versus pressure plot in Fig. 6B reveals that the pneu-net fabricated within the optimal bonding window was able to withstand greater operating pressures and achieve 50% higher curvatures than the pneu-net fabricated at τ_1_ greater than 3. Examination of failure mechanisms further reinforces this observation: The pneu-net with its components joined within the optimal bonding window fractured within the monolithic main body, while the pneu-net with components joined beyond their τ_1_^*^ failed because of delamination at the bonded interface. These results highlight the predictive power of the reaction coordinate framework to anticipate different failure modes and guide the fabrication process. By integrating our knowledge into the fabrication process, we demonstrate a path to improve the durability, performance, and life span of fully soft devices.

### Demonstrating strong layer-to-layer adhesion for additive manufacturing

**Fig. 7.**
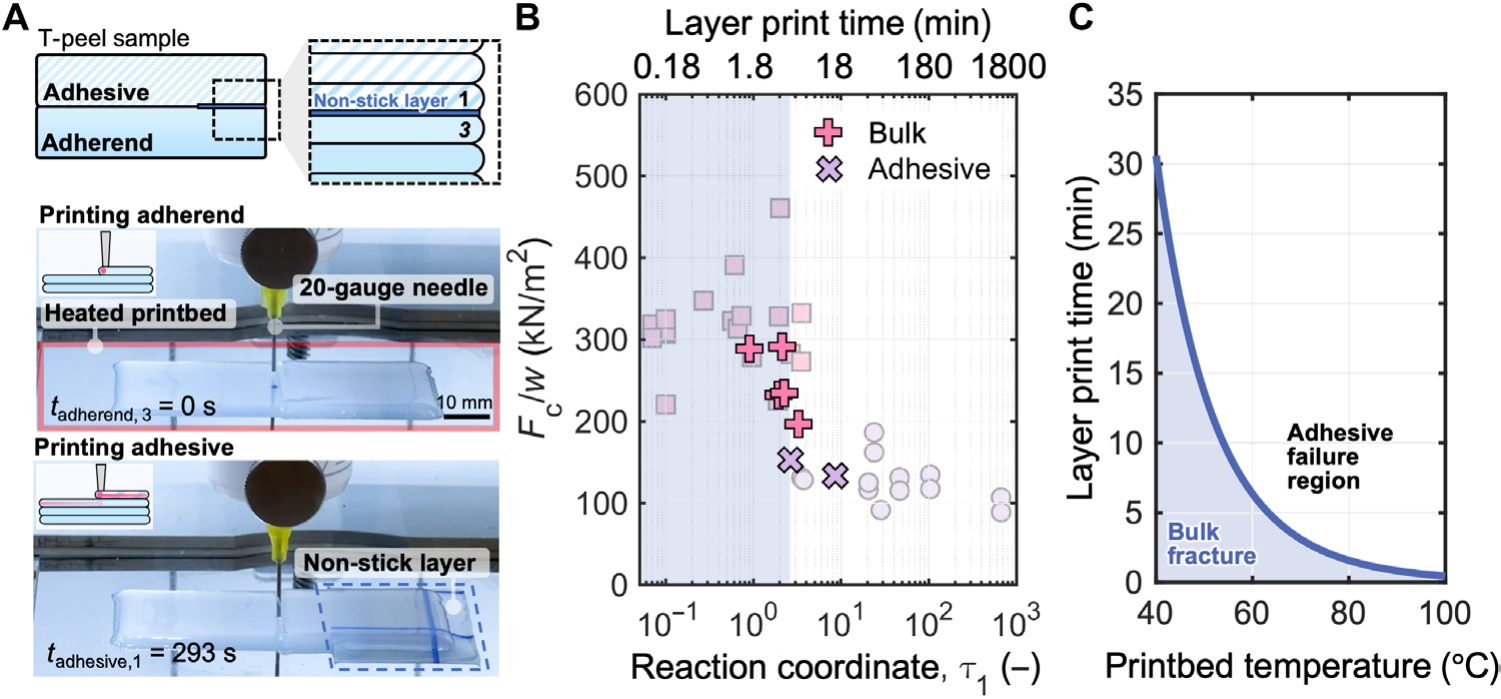
Tunable adhesion strengths for layer-by-layer printing. (**A**) DIW relies on the adhesion of ink to the preceding printed layer. We study the strength of the layer-to-layer adhesion for DIW of Ecoflex 00-30 by printing a T-peel geometry on a heated printbed and modulating the time between printing the final layer of the first T-peel arm and the first layer of the second T-peel arm (with each arm consisting of a total of three layers). A nonstick layer was manually introduced between the final layer of the first T-peel arm and the first layer of the second T-peel arm to define the peel arms. (**B**) T-peel results indicated by the “cross” symbols from the printed samples with varying τ_1_ values, overlaid onto T-peel results for thickness of 1.5 mm at room temperature (Fig. 3A), show that we can predict and control the mode of failure for DIW techniques. The upper horizontal axis shows durations between printing the final layer of the first T-peel arm and the first layer of the second T-peel arm, corresponding to the τ_1_ values in the bottom axis for printing at 65°C. The shaded blue region highlights the optimum printing window to achieve strong layer-to-layer adhesion. (**C**) The blue-shaded region in the plot highlights the bonding region for optimum layer print time to achieve strong adhesion as a function of printbed temperatures.

The findings from this work are not limited to tuning the adhesion strength between molded components but can also be extended to expand the design space for the additive manufacturing of silicone elastomers. During the DIW process of silicone elastomers, the layer-to-layer adhesion plays a substantial role in dictating the mechanical strength of the overall printed part. To demonstrate this concept, we used a 3D bioprinter and Ecoflex 00-30 as the ink to additively manufacture T-peel samples (Fig. 7A). The temperature of the bioprinter printbed was set to 65°C. For the printing parameters used (Materials and Methods), three layers of material were required to achieve the desired thickness of each arm of a T-peel sample. For this specific test and geometry, we identified that only the reaction coordinate at the interface between the final printed layer of the first T-peel arm (referred to as the adherend for consistency with the above-defined nomenclature) and the first printed layer of the second T-peel arm (referred to as the adhesive, although we reiterate that it consists of three layers) is crucial to the adhesion strength quantified in the T-peel test.

To predict the reaction coordinate τ_1_ during the application of the adhesive layer, we made two assumptions: (i) the prior printed layers are isothermal and achieve the set temperature of the printbed as soon as they are extruded as a conservative estimate, and (ii) because we chose a constant print speed and a repeating printhead path for each layer, offset only in the *z* direction, the duration at which any portion of the printed material is exposed to an elevated temperature should be the same upon deposition of the subsequent layer.

With these assumptions, we focused on tracking the time between printing through the midpoint of the final layer of the adherend and the first layer of the adhesive, which we call the layer print time. We intentionally vary the time in between printing the adherend and the adhesive to achieve different values of τ_1_, which are determined using Eq. 1. The upper horizontal axis shows the corresponding layer print times used to determine τ_1_ at 65°C. Between printing the adherend and adhesive layer, a nonstick layer was introduced to define the peel arms. The printed T-peel samples were then tested with the same parameters as in the previous experiments, with results for Ecoflex 00-30 shown in Fig. 7B (fig. S13 shows results for Dragon Skin 30). To account for the different thicknesses of the printed samples, we normalized the *F*_c_/*w* results by the actual measured thickness and normalized the experimental data from Fig. 3A for 1.5 mm by the thickness; we achieve comparable critical peel force values for both bulk and adhesive failure modes as indicated when compared to the molded T-peel samples (shaded data), and we are still able to predict the failure mode based on the reaction coordinate even for layer-by-layer manufacturing. The results also indicate that strong in-plane adhesion was achieved during the printing process (samples did not fail prematurely during the T-peel test; fig. S14). In Fig. 7C, we present guidelines for layer printing time as a function of printbed temperatures, using Eq. 1 and τ_1_^*^ as our target reaction coordinate, to ensure that the part was printed within the bulk fracture bonding region and has strong layer-to-layer adhesion. The interfacial strength between layers printed with τ_1_ < τ_1_^*^ was ~200% of the interfacial strength between layers printed with τ_1_ > τ_1_^*^, illustrating the importance of considering the reaction coordinate for printing strong materials and devices from silicone elastomers.

## DISCUSSION

We present a framework for studying the adhesion energy of commercially available platinum-catalyzed silicone elastomers that can be applied to arbitrary curing conditions, including time-varying curing temperatures, which are often used to accelerate curing either for faster throughput of molded components or to transition elastomers to their solid-like state after extrusion for higher-resolution DIW. The reaction coordinate serves as a useful tool to map out failure regimes and guide the design of robust elastomeric devices. We showed the failure mechanism using T-peel tests for two silicone elastomers (Ecoflex 00-30 and Dragon Skin 30); this framework can be extended to other thermally curable elastomers (*50*). We also explored the effect of sample thickness on the resulting critical peel forces, and we found that the critical peel force for bulk fracture is thickness dependent. The thickness also affects the value of the critical reaction coordinate τ_1_^*^, particularly for thin samples, because the force required to result in bulk fracture can be lower than the force needed to delaminate the interface. These findings highlight the importance of considering the thickness when studying the adhesion strength and could lead, in future work, to development of a modeling framework that allows for the prediction of critical reaction coordinate as a function of thickness. We showed that the adhesion energy itself, however, does not depend on the sample thickness, in line with physical intuition. A modeling framework incorporating the bulk fracture energy and adhesion energy could be beneficial to better predict τ_1_^*^ for varying thicknesses. We also verified that our modeling framework is able to predict the modes of failure even for elastomers exposed to time-varying heating conditions. Last, we demonstrated the applicability of the framework to real-world manufacturing techniques by fabricating pneu-net actuators at varying cure extents and taking advantage of an optimal bonding window identified from our modeling framework to achieve strong adhesion at the interface; furthermore, we showed the relevance to 3D printing of silicone elastomers, where accurately engineering the mode of failure can increase strength by 200%.

To establish our framework, we focused on only varying the temperature and duration of curing, and we kept the T-peel experiment procedures constant, including the rate of strain, which was quasi-static in all of our tests. Future work could characterize how faster strain rates during T-peel testing affect τ_1_^*^, which could be an important consideration when accounting for the rate of pressurization of pneumatic actuators or modeling rapid deformation of soft devices. The demonstrated framework allows for careful planning and designing of the fabrication process for elastomeric devices to ensure strong interfacial adhesion at joints and bonded regions, decreasing the likelihood of failure due to delamination and improving the overall yield. The accompanying demonstration of DIW with silicone elastomers could be further optimized to develop innovative technologies that allow for the precise control of curing kinetics and layer-to-layer adhesion based on the framework presented in this work. We expect that researchers in the field of additive manufacturing will use this understanding, in conjunction with the understanding of the temperature-dependent reaction kinetics, to develop algorithms that account for the extent of curing and the resulting adhesion strength between layers to optimize printing processes. The guidelines presented here, as well as broader efforts in the field to improve the adhesion of dissimilar materials (*21*), will broaden the design space and generate advanced capabilities in the manufacturing of soft, elastomeric devices.

## MATERIALS AND METHODS

### Fabrication of T-peel samples

We fabricated the T-peel test samples by first mixing equal masses of “part A” and “part B” of Ecoflex 00-30 and Dragon Skin 30 (Smooth-On) according to the manufacturer’s data sheet. The mixture was then degassed for 5 min for Ecoflex 00-30 and 15 min for Dragon Skin 30 to remove any trapped air bubbles. To achieve the desired sample thickness, we calculated the requisite mass of prepolymer poured into the 3D-printed mold on the basis of the density and volume required to fill half of the mold. Transfer tape (Vinyl Ease) was used as the nonstick layer to define the peel arms: After fabricating the first layer, a 30 mm–by–40 mm section of transfer tape was placed onto the supporting tabs of the 3D-printed mold. The adhesive on one side of the transfer tape allows adhesion to the tabs, providing support holding it in place. Then, the second layer of prepolymer was poured into the mold to create the adhesive layer, at which point the reaction coordinate τ_1_ was recorded.

### Testing of T-peel samples

We perform the T-peel tests on a universal testing machine (Instron, 68SC-2). The samples were trimmed down to a width of 25 mm to eliminate edge effects caused by the formation of menisci along the edges of the mold walls. The nonstick layer was removed, and the peel arms were secured in the pneumatic grips of the universal testing machine at a pressure of 100 kPa. The experiment was performed at a quasi-static strain rate of 100 mm/min, and the resulting force was recorded as a function of displacement with a 2-kN load cell.

### Pneu-net fabrication

Degassed prepolymers were poured into 3D-printed negative molds and cured to fabricate the main bodies of pneu-net actuators (fig. S10A). Once the desired τ_1_ of the actuator body was achieved, a strain-limiting rectangular base layer was fabricated by placing inextensible muslin fabric on the bottom of the base and pouring freshly mixed prepolymer onto the inextensible layer (fig. S10B). The actuator body was then placed onto the base, and the entire actuator was allowed to fully cure. An 18-gauge needle was used to create an air inlet hole near the base of the actuator body, and a silicone tube with a ^1^/_16_-inch outer diameter (McMaster-Carr, 5236K204) was inserted and glued in place with RTV silicone adhesive (Momentive, RTV 103) to create the fluidic connection. During the experiment, pneu-net actuators were connected to a syringe pump at a flow rate of 200 ml/min. The input pressure was measured using a pressure sensor (Panasonic, ADP5151) with an analog voltage acquisition device (National Instruments, USB-6002). All data acquisition, processing, and plotting routines were performed in MATLAB.

### 3D printing of T-peel geometries

We used a bioprinter (CELLINK, BIO X) to study the adhesion between layers as a function of the reaction coordinate for additive manufacturing of silicone elastomers. The bioprinter was equipped with a heated printbed and a pressure-controlled pneumatic printhead. The printing was conducted at 100 kPa, with a print speed of 15 mm/s, with a printbed temperature of 65°C (the maximum printbed temperature of the printer), and using 20-gauge needles. The ink (Smooth-On, Ecoflex 00-30) was prepared by premixing the prepolymer and loading it into a 3-ml syringe (fig. S16). Because of the low volume capacity of the syringe, we decreased the overall size of the T-peel geometry to 55 mm by 28 mm by 1.5 mm. The geometry was designed in SolidWorks, and the stereolithography (STL) file was converted into G code using an open-source slicer software (Slic3r). We chose a 30% infill for Ecoflex 00-30 to accommodate spreading of the elastomer in its liquid-like state before curing. We also printed T-peel samples using Dragon Skin 30 (fig. S13). Because of the heated printbed, the elastomer cures and solidifies within a relatively short time and therefore experiences limited spreading after extrusion, which is desirable in terms of good resolution of printed parts. To calibrate the printer, we zeroed the printhead at the center of the printbed. The center of the printbed was marked (indicated with the black dot on the printbed in Fig. 7A) to serve two purposes: (i) to allow for consistent printing location of the subsequent adhesive layer and (ii) to track the duration (manually, using a stopwatch) between printing the final layer of the first T-peel arm and the first layer of the second T-peel arm, to predict the τ_1_ value. Immediately after the final (third) layer of the first T-peel arm was printed, a nonstick layer was introduced on one end to define the peel arms. To create samples with low τ_1_ values, printing was then immediately resumed (to limit the layer print time), and the second T-peel arm was printed. The printhead was zeroed at the center marker on top of the previously printed layer. For samples with high τ_1_ values, after some layer print time, the syringe was reloaded with freshly mixed prepolymer to ensure that τ_2_ = 0, and the second T-peel arm was printed. The samples were trimmed at the edges to avoid edge effects during T-peel testing, and the final width and thickness of the samples were measured.

### Temperature measurements of elastomer during curing

The real-time temperature measurements of the elastomer and the oven were obtained using 0.01-inch-diameter fiberglass-insulated T-type thermocouples from Omega. The thermocouple was slotted into one of the 3D-printed T-peel sample mold. Temperature data were processed using MATLAB with an NI-9212 data acquisition (DAQ) device.

## Supplementary Materials

### The PDF file includes:

Supplementary Text Figs. S1 to S16 Tables S1 and S2 Legends for movies S1 and S2 References
