# Supplementary material for: Understanding silicone elastomer curing and adhesion for stronger soft devices
Source: Sci Adv. 2025 Jul 16;11(29):eadv2681. doi: 10.1126/sciadv.adv2681 (PMC12266107; doi:10.1126/sciadv.adv2681)
Supplement: Supplementary file 1 — Supplementary Text Figs. S1 to S16 Tables S1 and S2 Legends for movies S1 and S2 References [file sciadv.adv2681_sm.pdf]

Science Advances
AAAS

Supplementary Materials for

Understanding silicone elastomer curing and adhesion for stronger soft devices

Te Faye Yap *et al.*

Corresponding author: Daniel J. Preston, djp@rice.edu

Sci. Adv. **11**, eadv2681 (2025)
DOI: 10.1126/sciadv.adv2681

The PDF file includes:

Supplementary Text
Figs. S1 to S16
Tables S1 and S2
Legends for movies S1 and S2
References

Other Supplementary Material for this manuscript includes the following:

Movies S1 and S2

Supplementary Text

Determination of E_a and ln(A) for platinum-catalyzed silicone elastomers

The values of activation energy and frequency factor for platinum-catalyzed silicone elastomers were determined with small amplitude oscillatory shear (SAOS) tests performed to characterize the rheological properties of the elastomers during curing, as detailed in prior work *(50)*. SAOS tests were performed on an ARES G2 rheometer to track the dynamic moduli (i.e., the storage modulus G′ and the loss modulus, G″) of the elastomer during isothermal curing over time. The curing temperature was controlled using a Peltier module. A cone-and-plate geometry was used to perform the SAOS test. The angular frequency and strain amplitude was 10 rad/s (or 1.6 Hz) and 0.25 %. 1 mL each of part A and B were mixed and dispensed onto the preheated geometry. The results from the SAOS tests allowed us to determine the gelation point which is the time at which G″ = G′. The gelation time includes the setup time which accounts for the time it takes to dispense the elastomer on the geometry, to trim the edges, and move to the geometry gap for testing. The tests were performed at four different temperatures, and the gelation time t_{gel} at each temperature was determined. Each pair of values of t_{gel} and T was plotted according to the linearized Arrhenius equation:

$$\ln(t_{gel}) = \frac{E_a}{RT} - \ln(A) \qquad Eq. S1$$

The slope and intercept of the linear fit were used to determine the physical parameters E_a and ln(A). Fig. S1 illustrates an example of the methodology used for determining E_a and ln(A) for Ecoflex 00-30. Table S1 tabulates the E_a and ln(A) values determined Ecoflex 00-30 and Dragon Skin 30 that were determined from our prior work, and are used in this study. These values can be used in Eq. 1 in the main text to determine the nondimensional reaction coordinate τ_1.

T-peel sample molds

The T-peel samples of different thicknesses were fabricated by dispensing well-mixed *(50, 59)* prepolymer into 3D printed molds (Fig. S2). The molds were printed using a masked stereolithography (MSLA) printer (Peopoly Phenom with Siraya Tech Fast Resin). Unreacted resin species often act as cure inhibitors of platinum-catalyzed silicone elastomers; therefore, to circumvent this source of uncertainty, a thin layer (< 10 μm) of lacquer (Rust-Oleum) was sprayed onto each mold to passivate the surface. The mold was intentionally designed to be wider than the desired final specimen and we included raised guides that were 7.5 mm away from the side walls to guide trimming and removal of the edges to account for edge deformation due to the formation of a meniscus in the liquid prepolymer and to ensure the final width of each T-peel sample was consistent. Tabs corresponding to the desired thickness of each layer were included to support the non-stick layer that was used to create the peel-arms. An opening at one end of the mold was introduced to accommodate the thermocouple and measure the temperature of the first layer (adherend) as it was curing. The final dimensions of a single layer of the T-peel samples are 100 mm by 25 mm by 1.5, 2.5, or 4 mm with a peel arm length of 30 mm, which are comparable to the ASTM D1876 geometry (*60*).

T-peel sample fabrication and testing

To determine the failure mode transition reaction coordinate, we used a coarse-to-fine analysis approach by fabricating a large array of samples with varying τ_1 values (typically around the

gelation point and cure point of the elastomer), and once we observe a change in failure mode, we narrow our search and focus our sample fabrication within the transition regime. The samples were also fabricated at room temperature to serve as a baseline. To minimize error, we monitored the temperature of the samples using a thermocouple through the thermocouple insert designed into the T-peel sample molds. When calculating the reaction coordinate based on constant ambient conditions, it is important to determine the average ambient temperature. We show that ±3 °C would result in an increase in 32% a decrease in 24% from 23°C when calculating the reaction coordinate for 26 °C and 20 °C, respectively, which would result in different modes of failure close to the critical transition regions. We also performed an analysis showing that the average room temperature can be used as a proxy for sinusoidal temperature fluctuations with minimal deviation which would result in less than 3% and 7% deviation for temperature fluctuations of ±3 and 5°C with frequencies of 10 minutes and 1 hour per cycle (Fig. S3C-F). If a constant room temperature condition is considered, Eq.1 can be simplified to a linear equation as shown in Eq. S2:

$$\tau(t) = Ae^{-\frac{E_a}{RT}}t + \tau_0 \qquad Eq.S2$$

For cases where elevated temperatures are employed (i.e., heating in a furnace to accelerate curing), a thermocouple would be required to accurately monitor the sample temperature during heating, because the sample's thermal mass introduces a transient heating period before reaching thermal equilibrium with the furnace environment. In scenarios where thermocouples may not be accessible or practical, using the furnace setpoint temperature in the model provides a conservative approach to estimating the reaction coordinate. By comparing the reaction coordinate computed under the assumption of a constant furnace setpoint temperature with that determined by modeling the transient temperature rise using a lumped capacitance system (Eq. S3), the furnace setpoint overpredicts the actual reaction coordinate at a given time (Fig. S4A & B). This conservative approach ensures that bonding can be performed prior to reaching the critical transition reaction coordinate τ_1^*, allowing strong interfacial adhesion while still taking advantage of accelerated curing.

$$T(t) = (T_i - T_o)\exp\left(-\frac{hA_s}{\rho C_p V}t\right) + T_o \qquad Eq.S3$$

Fig. S5 shows the fabrication process for a T-peel sample. Part A and Part B for a given elastomer was mixed at a 1:1 ratio and subsequently degassed for 5 and 15 minutes respectively for Ecoflex 00-30 and Dragon Skin 30. The mixed prepolymer is then poured into the T-peel molds by weight to achieve the specific thicknesses, and the MATLAB script recording the real-time temperature was initiated. Simultaneously, the recorded temperature is used to calculate real-time reaction coordinate of the elastomer. Next, we place a non-stick layer (Transfer Tape, VinylEase) to create the arms of the T-peel sample. To ensure that the non-stick layer alignment is repeatable and perpendicular, we fabricate the non-stick layers using a CNC vinyl cutting machine (Maker, Cricut USA) to ensure the dimensions of the rectangular non-stick layers are consistent and we used the tabs designed in our mold for alignment and support. A second batch of prepolymer is mixed and degassed to create the adherend, and the value of τ_1 during pouring

of the second layer onto the first was recorded (according to the real-time reaction coordinate) for the specific sample.

Once both layers of the sample have fully cured, both lengthwise edges of the samples were trimmed according to the recessed imprint on the samples from the raised guides using a guillotine paper cutter to eliminate effects that arise from the menisci that form on the edge of the mold. The final width of the sample is 25 mm (similar to the width of the grips on the universal testing machine). The non-stick layer was removed, and the sample is loaded into the pneumatic grips of the universal testing machine (68SC-2, Instron). When securing the sample, we aligned the width of our sample parallel to the edges of the pneumatic grips. The samples were loaded at strain rate of 100 mm/min, and the resulting force was a recorded with a 2 kN load cell as a function of displacement.

Determining the critical peel force-per-width values from T-peel test data

To determine the critical peel force-per-width for T-peel samples that undergo adhesive failure, we analyzed the force-versus-displacement curves by identifying key features using the *findpeaks* function in MATLAB. The plateau region was defined between the index of the first peak and final detected peak. To avoid capturing noise at the onset of peeling or edge effects near the end of the sample, only the central 80% of the steady-state peeling region was selected for averaging, symmetrically truncating 10% from both ends of the plateau region. Fig. S6 shows an example of force-per-width versus displacement plot for a sample undergoing adhesive failure. The bounded region in Fig. S6 represents the 80% of data that was used to determine the average plateau force. This method provides a consistent and standardized approach to quantifying adhesive strength across all the sample data. As for samples that undergo bulk fracture, the critical force-per-width value was determined as the maximum force-per-width experienced by the sample before fracture.

Probability of mode of failure as a function of adherend reaction coordinate

To determine the probability of a single mode of failure, we used logistic regression to model the binary failure outcomes (bulk fracture = 1, adhesive failure = 0) as a function of the τ_1. This approach allows us to estimate the probability of bulk fracture across a continuous range of τ_1 values based on our experimental data. We performed the logistic regression with MATLAB, using the *fitglm* (generalized linear regression model) function with a binomial distribution to model the probability of bulk fracture, according to the logistic curve:

$$P(bulk\ fracture) = \frac{1}{1 + e^{-(\alpha + \beta \tau_1)}} \qquad Eq. S4$$

Where α and β are the intercept and slope determined from the regression fit. To visualize the transition from bulk fracture to adhesive failure mode, we plotted the probability of bulk fracture as a function of τ_1 for all our experimental data (Fig. S7), which include data for Ecoflex 00-30 (t = 1.5, 2.5, and 4.0 mm) and Dragon Skin 30 (t = 2.5 mm). To determine key transition values, we rearranged Eq. S4 to determine τ_1 values for the case of 95%, 50%, and 5%:

$$\tau_1 = \frac{\ln\left(\frac{P}{1-P}\right) - \alpha}{\beta} \qquad Eq. S5$$

We chose to highlight threshold probabilities of 95%, 50%, and 5%, with 95% indicating a high likelihood of bulk fracture, 50% marking the transition region, and 5% indicating a strong likelihood of adhesive failure. We tabulated the three probability values for each experiment in Table S2. In the main text, we indicate the τ_1^* as the τ_1 value that corresponds to a 50% probability of bulk fracture as a median transition point.

Finite element simulation of bulk fracture and adhesive failure modes

Numerical simulations were conducted using the commercial finite element software SIMULIA Abaqus 6.14-1. The constitutive material model for Ecoflex 00-30 was first calibrated using tensile test data. Fig. S8A illustrates the dogbone sample geometry, which was modeled as a plane stress problem using CPS4R elements—a 4-node bilinear plane stress quadrilateral with reduced integration and hourglass control. The sample geometry and dimensions were consistent with those used in the experimental tests. The material behavior of Ecoflex was modeled using the Yeoh model, and the uniaxial tensile test data were employed to calibrate the constitutive constants. Fig. S8B presents a comparison between the numerical and experimental nominal stress vs. engineering strain data, with Fig. S8C showing the axial stress distribution at various stages of deformation. We note that this numerical model does not account for material failure, as no damage modeling was incorporated, and thus the stress during failure could only be estimated based on experimental results. From the experimental results, we determined the failure point occurs at engineering strain of 14 mm/mm, and we can correlate the strain to stretch using Eq. S6:

$$\lambda = \varepsilon_{eng} + 1 \qquad Eq. S6$$

Fig. S8D shows the ratio of true stress to nominal stress as a function of stretch. At the failure point, this ratio was approximately 15, indicating that the true stress during failure is approximately 15 times greater than the nominal stress (1.38 MPa). This value corroborates the FEM results we highlight in Fig. 3B, where we compare the forces required to achieve cohesive fracture for different thicknesses, indicating that each sample thickness experiences the same stress during failure.

To model the T-peel test, a finite element model was developed using a 2D plane strain assumption with CPE4R elements, which are 4-node bilinear plane strain quadrilaterals with reduced integration and hourglass control. Fig. S9A shows the 2D T-peel FE model and geometry used in the FEM analysis, where the inset shows the blowup of the mesh near the interface with refined mesh size. The geometric dimensions of the T-peel samples were consistent with the experimental design. Fig. S9A shows the finite element model of the T-peel sample with a thickness of 2.5 mm. A quasi-static analysis was performed, stabilized with a slight damping factor of 5×10^{-3} for energy dissipation. The mesh had an average size of 0.1 mm, refined near the interface to an element size of approximately 0.02 mm.

Two models were utilized: one to explain the failure forces experienced in the case of bulk fracture and the other to predict interfacial adhesion energy and calibrate the adhesion energy equation during adhesive failure. In the first model, the interface was treated as a perfect interface using a tie contact. The failure force for bulk fracture was estimated when the true stress in the upper arm, near the interface, reached the critical true stress level obtained from the

tensile test (Fig. 3B). We demonstrate that all three thicknesses experience similar stress values near the bonding interface, with $\sigma_{22} \approx 18$ MPa during failure. This value closely resembles the true tensile strength of the material, determined by modeling the axial tensile stress of the same material using FEM, and correlating it using the stretch of the material during failure, with the ultimate tensile strength reported by the manufacturer's datasheet.

In the second model, the adhesive failure model utilized surface-based cohesive contact with a triangular traction-separation curve as shown in Fig. S9B. For a sample thickness of 2.5 mm and a high reaction coordinate, the parameters of the cohesive traction-separation curve—initial stiffness (k), critical stress for damage initiation ($\hat{\sigma_c}$), and fracture toughness (γ)—were calibrated by matching the experimentally obtained force-displacement curves. We performed calibration as follows: starting with the adhesion energy measured from experiments, in the vicinity of 0.8-1.0 kJ/m², the initial slope of the traction-separation curve was calibrated using an interface strength to replicate the initial slope of the force-displacement curve. The critical strength was then fine-tuned to match the steady-state force responsible for delamination at the interface. For this thickness, the cohesive parameters were determined to be $k = 10\ MPa/mm$, $\hat{\sigma_c} = 0.25\ MPa, \gamma = 0.8\ kJ/m^2$.

Thicker and thinner T-peel samples with high reaction coordinates were also modeled using these cohesive parameters, and the finite element model successfully reproduced the experimental force-displacement curves (Fig. 4B). Consequently, we concluded that for very high reaction coordinates, the interfacial toughness is independent of sample thickness. To match other levels of cohesive energy (from 0.8 to 2.0 kJ/m²) and calibrate the adhesion energy estimation equation (Eq. 6), the cohesive toughness was adjusted to align with the analytically estimated force-per-width (F/w) level for a given adhesion energy. The critical cohesive strength was scaled proportionally to the square root of the cohesive toughness ratio to ensure that the softening portion of the traction-separation curve remained self-similar.

To determine the effective modulus E^*, we employed a FEM to calculate the force per unit width responsible for interfacial adhesive failure across a range of adhesion energies (0.8–2.0 kJ/m²) and sample thicknesses (1.5 mm, 2.5 mm, and 4 mm) used in this study (Fig. 4C). The analytical equation (Eq. 6) was then fitted to the data points obtained from the FE analysis by adjusting the value of E^* for various adhesion energies. The E^* and Eq. 6 were then used to calculate the adhesion energy in Fig. 4D

Fabrication of pneu-net mold

The negative molds for the pneumatic network actuator (Fig. 6, Fig. S10) were fabricated using a fused deposition modeling (FDM) additive manufacturing process. The molds were designed using SolidWorks, and a Bambu Lab X1 Carbon FDM 3D printer was used to print the molds. The molds were printed from polylactic acid filament at an initial print speed of 50 mm/s for the first layer, a speed of 100 mm/s for the outer walls and infill, and print speeds of 200 mm/s and 300 mm/s for the outer and inner walls, respectively, on a textured PEI plate. The nozzle and build plate temperature were set to 220°C and 50 °C, respectively. The molds were removed from the build plate after the prints completed.

Effect of mold release spray on failure behavior

Elastomeric components that are cast in molds often rely on the use of mold release agent to facilitate the demolding process. However, these release agents can inhibit or weaken adhesion by creating a lubricant barrier at the surface. To mitigate this undesired effect, we can selectively apply mold release on areas that are essential for demolding, carefully avoiding the adhesion sites. To validate that mold release applied adjacent to bonding regions does not affect the failure behavior, we fabricated T-peel samples by spraying mold release to fabricate the peel arms, instead of using the transfer tape as a non-stick layer. The first layer of prepolymer was poured into the mold, with masking applied to protect the intended bonding areas, while mold release agent (Ease Release 200) was sprayed onto regions where the transfer tape would normally be used (the bonding region was masked), following by the adherend layer. T-peel samples with $\tau_1 = 2.5$ and $\tau_1 \gg 3$ were fabricated to examine the failure modes and critical force-per-width values. The findings shown in Fig. S11 demonstrate that applying the mold release agent adjacent to the bonding region does not affect the adhesion strength, and that we are able to use a mold release spray, while achieving strong and predictable adhesion, as long as the adhesion sites are not contaminated.

Effect of adhesive reaction coordinate τ_2 on critical force-per-width

Results in the main text primarily focused on T-peel samples that were fabricated with $\tau_2 = 0$, which includes a standard degassing period (either 5 or 15 minutes, depending on the elastomer used), and we only varied the adherend cure extent τ_1. To investigate how the reaction coordinate of the adhesive τ_2 influences the transition reaction coordinate $\tau_1^*$and the adhesion strength, we fabricated T-peel samples with varying τ_2 using Dragon Skin 30 (t = 2.5 mm). We selected τ_2 values of 0.25 and 0.50 to simulate practical conditions where the prepolymer remains pourable (lower viscosity), minimizing the risk of introducing bubbles during handling and ensuring a smooth surface during application. A third value, $\tau_2 = 2.5$, was chosen to represent a scenario where the adhesive has passed the gelation point and was solid-like and can be demolded and bonded within the optimal bonding window (Fig. 6). We fabricated a total of 21 samples across seven combinations of τ_1 and τ_2 (n = 3 for each combination).

Fig. S15 shows the results overlaid on data from Figure 2B. The results indicate that varying τ_2 does not have a substantial effect on the adhesion strength as the critical force-per-width values were comparable to the baseline ($\tau_1 = 0$), even for the case when both τ_1 and$\tau_2 = 2.5$. While the transition reaction coordinate τ_1^* showed a slight leftward shift for higher τ_2 values, the change falls close to the 95% probability band for transition to adhesive failure as predicted by logistic regression (Fig. S7). These results suggest that the adhesion behavior is relatively insensitive to changes in the adhesive's reaction at the time of bonding. This observation is relevant for applications such as multi-component assembly of elastomeric devices or direct ink writing, where the adhesives may experience partial curing prior to bonding. The results support the robustness of our framework

Predicting the failure modes for multi-component assemblies

To study if our framework can be applied to cases where multiple components are separately cast and assembled, we fabricated T-peel samples with two adherends τ_1 that were bonded together with adhesive τ_2. We fabricated these samples using Dragon Skin 30, because Dragon Skin 30 has an optimal bonding window (Figure 6) which allows us to demold the parts for assembly and

achieve both bulk fracture and adhesive failure modes. Two combinations of samples were fabricated: (i) $\tau_1 = 2.5$ and (ii) $\tau_1 \gg 3$. The results in Fig. S16 indicate that we are able to predict the failure modes as well as achieve a strong interface during bonding if the reaction coordinate of the adherends is lower than τ_1^*.

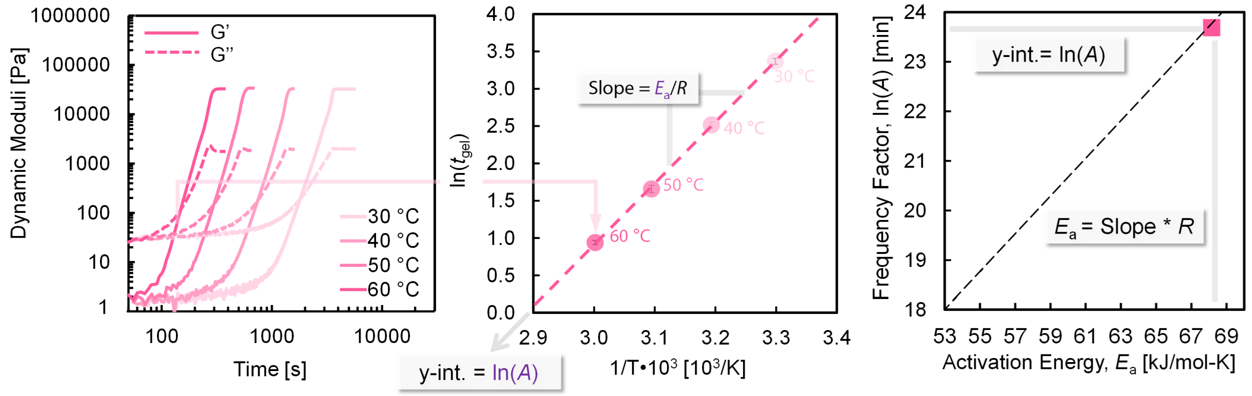

Fig. S1. Methodology for determining E_a and ln(A) for Ecoflex 00-30 and Dragon Skin 30. Dynamic moduli curves were determined from small amplitude oscillatory shear (SAOS) tests at various temperatures (data for Ecoflex 00-30 is shown here). The gelation time (i.e., the crossover point when the storage and loss moduli curves intersect) at each temperature is then plotted according to the linearized Arrhenius equation. The slope and intercept were used to back-calculate the activation energy and frequency factor.

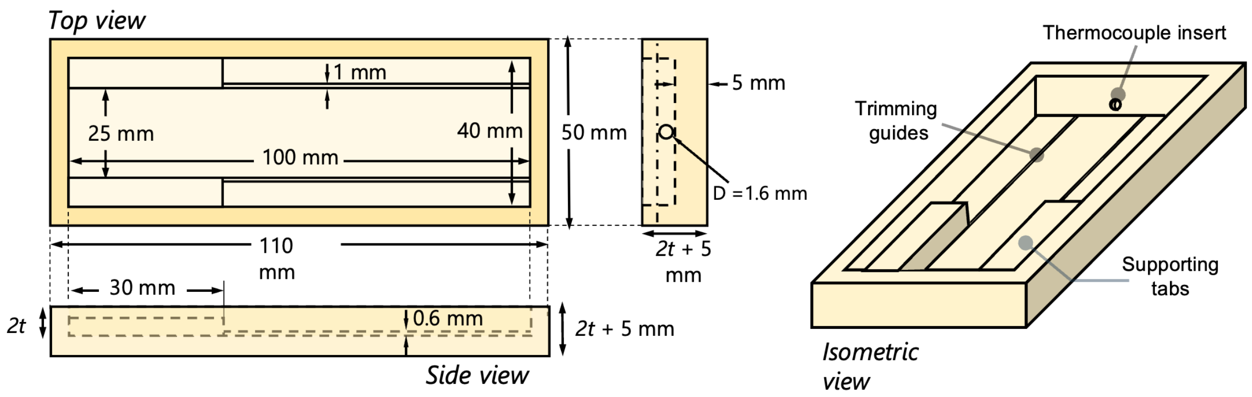

Fig. S2. Dimensions of mold used to fabricate T-peel sample. The t represents the different thicknesses studied in this work, where t = 1.5 mm, 2.5 mm, and 4.0 mm. Additional tabs at one end of the mold support the non-stick layer after the first layer of the elastomer is poured.

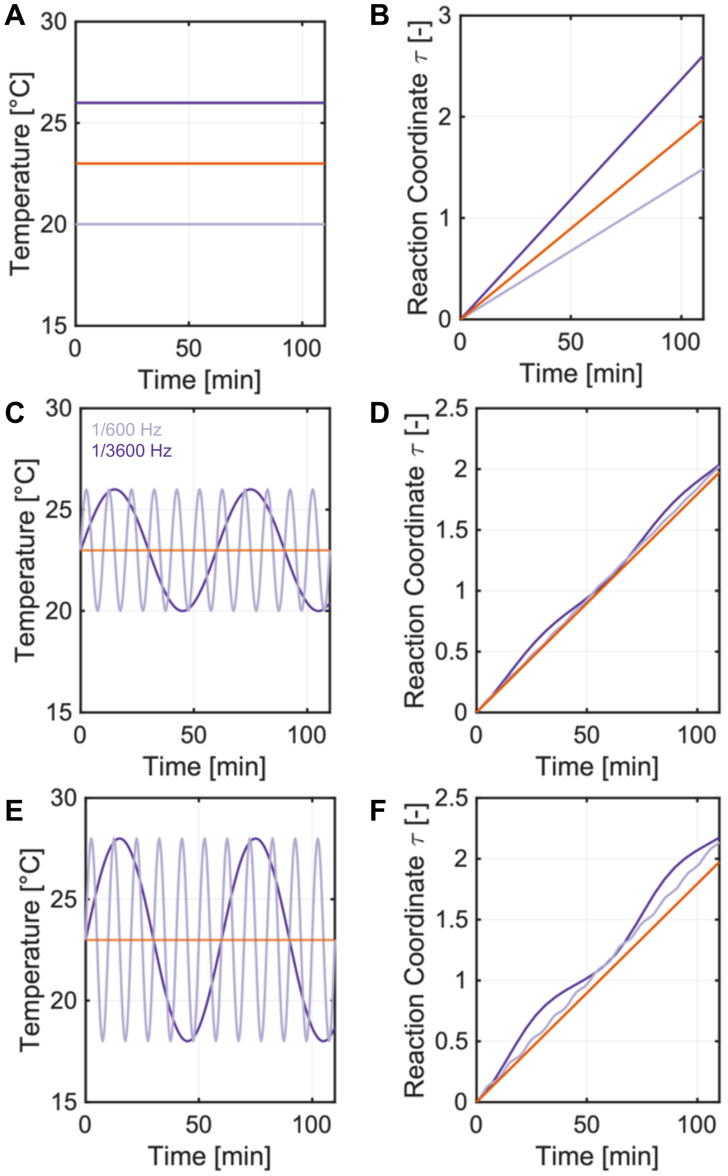

Fig. S3. Effect of time-varying temperatures on the rate of curing for ambient conditions. (A & B) Cure extent as a function of time for 20, 23, and 26 °C. A ±3 °C deviation from 23 °C results in a 32% increase and 24% decrease in reaction coordinate for 26 °C and 20 °C respectively. (C & E) Sinusoidal temperature profiles to simulate temperature fluctuations ±3 and 5°C around the average room temperature (23°C). The simulated temperature profiles have frequencies of 1/600 Hz and 1/3600 Hz which correspond to 10 minutes and one hour per cycle. (D & F) Corresponding reaction coordinate of the elastomer as a function of time and temperature. The model shows that temperature fluctuations result in a less than 3% and 7% percentage difference in reaction coordinate upon approaching cured conditions.

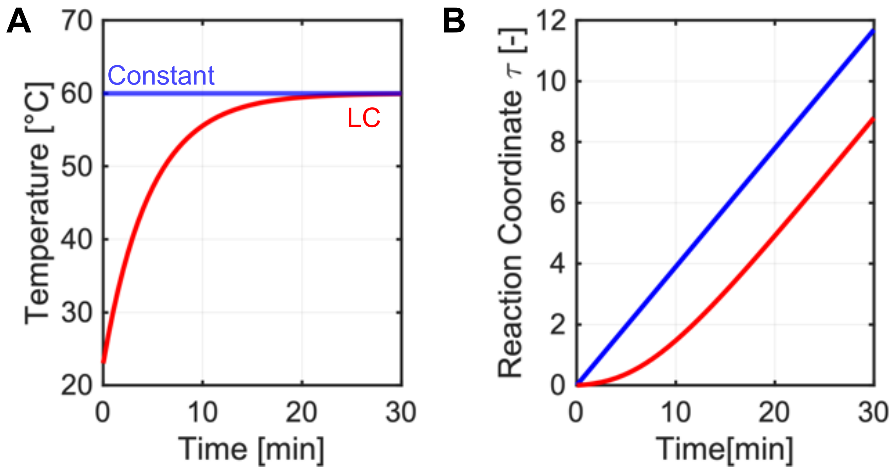

Fig. S4. Elevated temperature curing in an oven. (A) Example temperature profiles for a sample undergoing elevated temperature curing in a furnace and setpoint temperature. The simulated furnace setpoint temperature was 60°C, and the model assumes that the sample heating conditions follow the lumped capacitance model (which would change the slope of the initial temperature rise). (B) The reaction coordinate results indicate that using the furnace setpoint temperature during modeling would result in a higher reaction coordinate for the same heating duration, making it a conservative estimate.

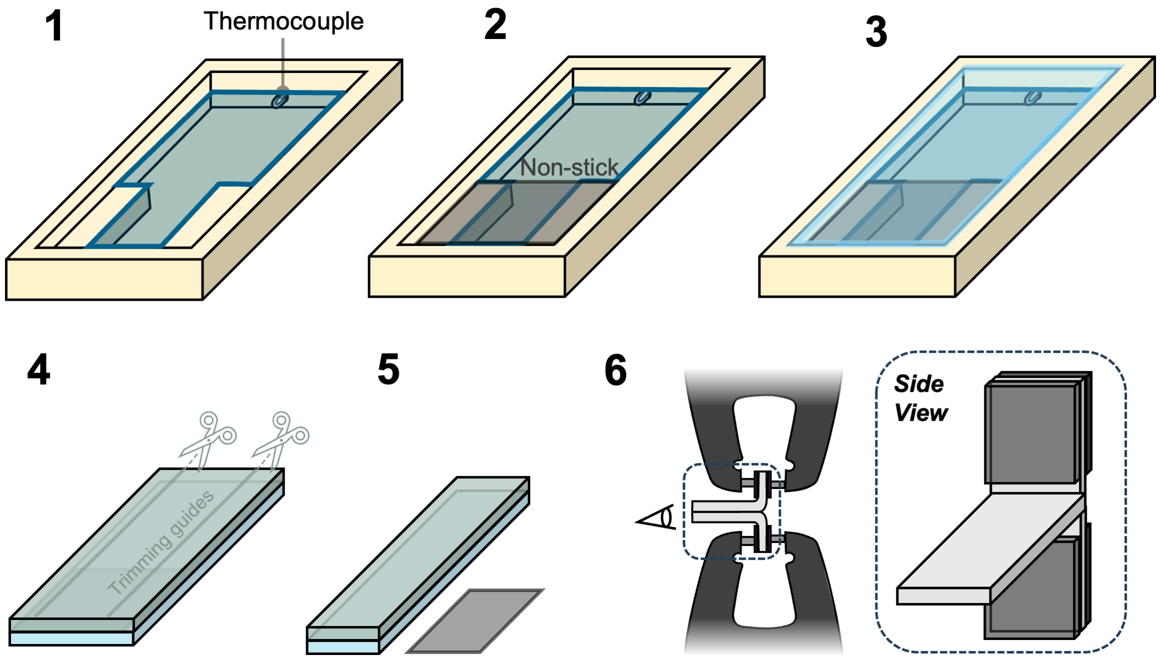

Fig. S5. T-peel test sample fabrication procedure. (1) A thermocouple is used to record the real-time temperature of the adherend (layer 1) during curing. (2) A non-stick layer is aligned on the tabs and the edges of the mold to create the peel arms. (3) The adhesive layer (layer 2 following the convention in the main text) is poured onto the adherend layer, and the reaction coordinate τ_1 is recorded. (4) The samples are allowed to fully cure, and the edges are trimmed according to the trimming guides. (5) The final trimmed sample has a width of 25 mm, and the non-stick layer is removed. (6) The T-peel samples are secured to the universal testing machine with pneumatic grips. The peel arms are aligned with the edges of the pneumatic grips as indicated by the side view illustration.

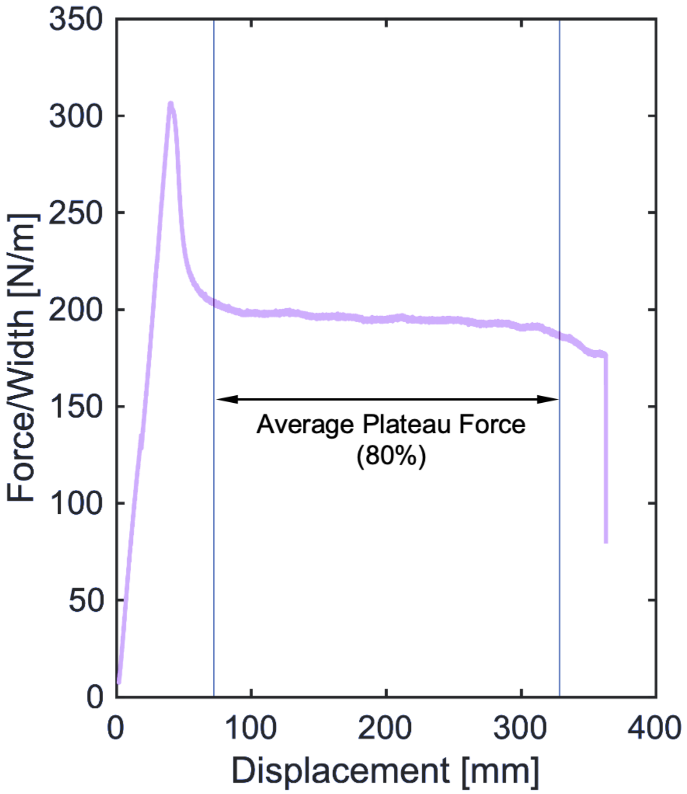

Fig. S6. Determining the average plateau force for samples undergoing adhesive failure. The average plateau force is determined from 80% of steady state peeling regime, truncating 10% of data from the peak of both ends symmetrically according to the peak analysis described above.

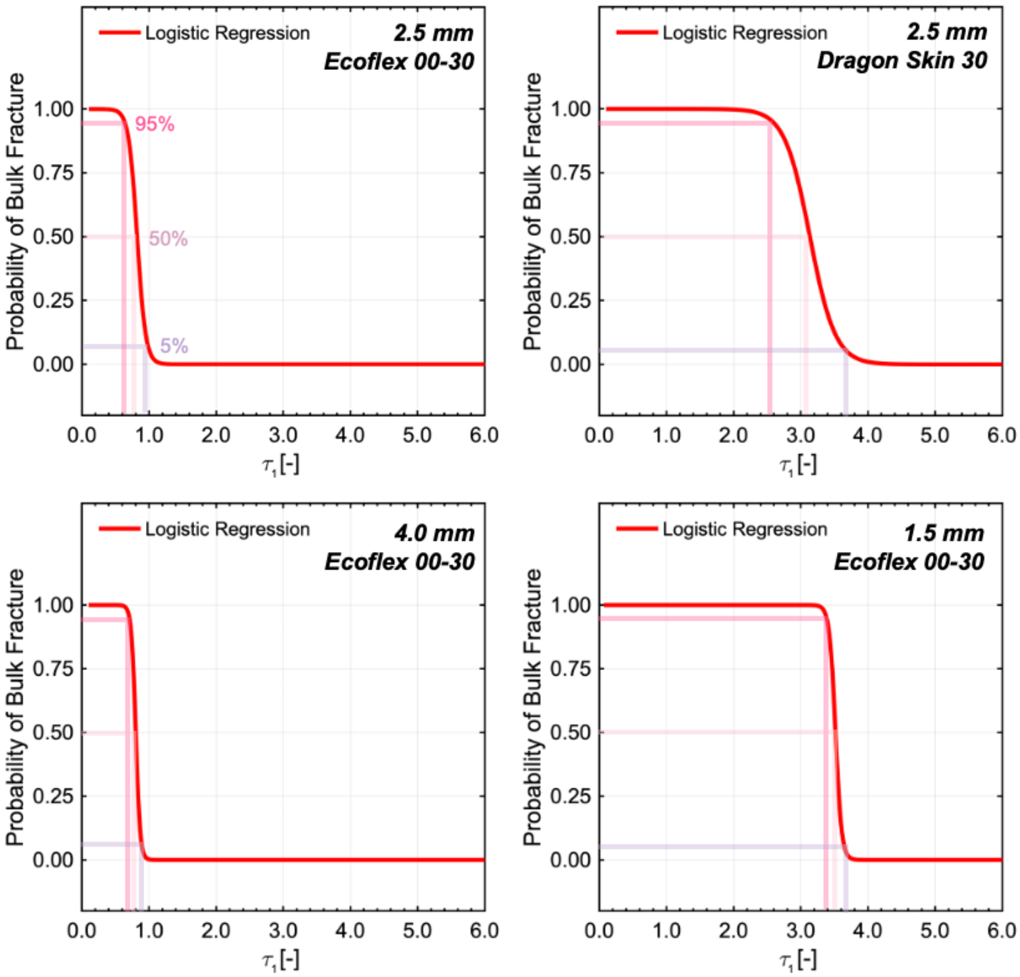

Fig. S7. Probability of bulk fracture determined using logistic regression. The probability of bulk fracture occurrence plotted as a function of τ_1. τ_1 values for 95%, 50%, and 5% probability of bulk fracture are highlighted in the plots as well as in Fig. 2.

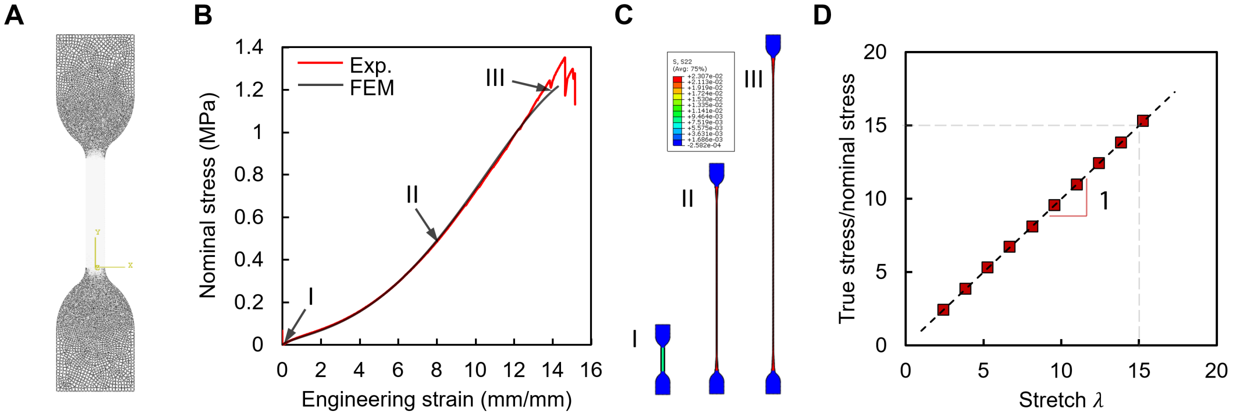

Fig. S8. Calibration of FEM variables based on experimental tensile test data. (A) Dogbone sample geometry used in the FEM analysis. (B) Comparison of experimental and simulated engineering stress-strain curves for the tensile test of Ecoflex 00-30. (C) Axial stress distribution at various stages of deformation. (D) Ratio of true stress to nominal stress as a function of stretch, illustrating that true stress is proportional to nominal strain, with the stretch acting as the proportionality constant.

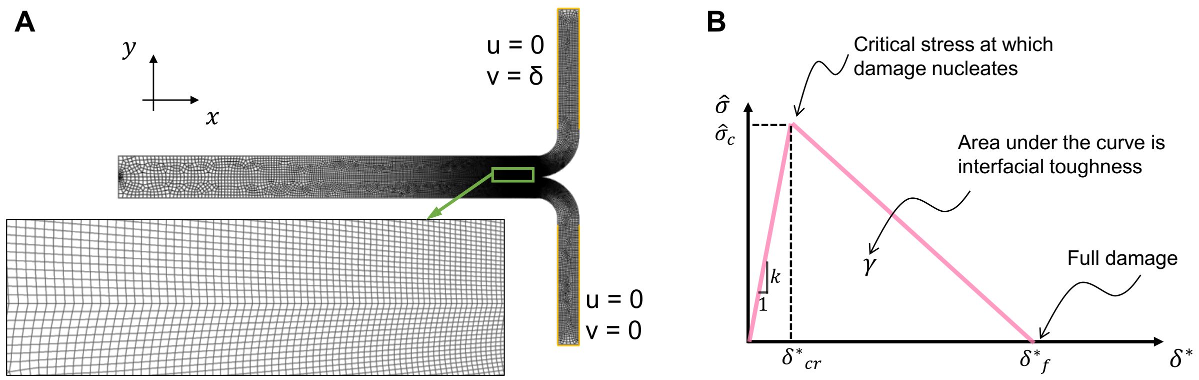

Fig. S9. Schematic of model used to analyze adhesive failure during T-peel tests. (A) 2D T-peel FE model and geometry used in the FEM analysis, where the inset shows the blowup of the mesh near the interface with refined mesh size. (B) Triangular traction-separation curve.

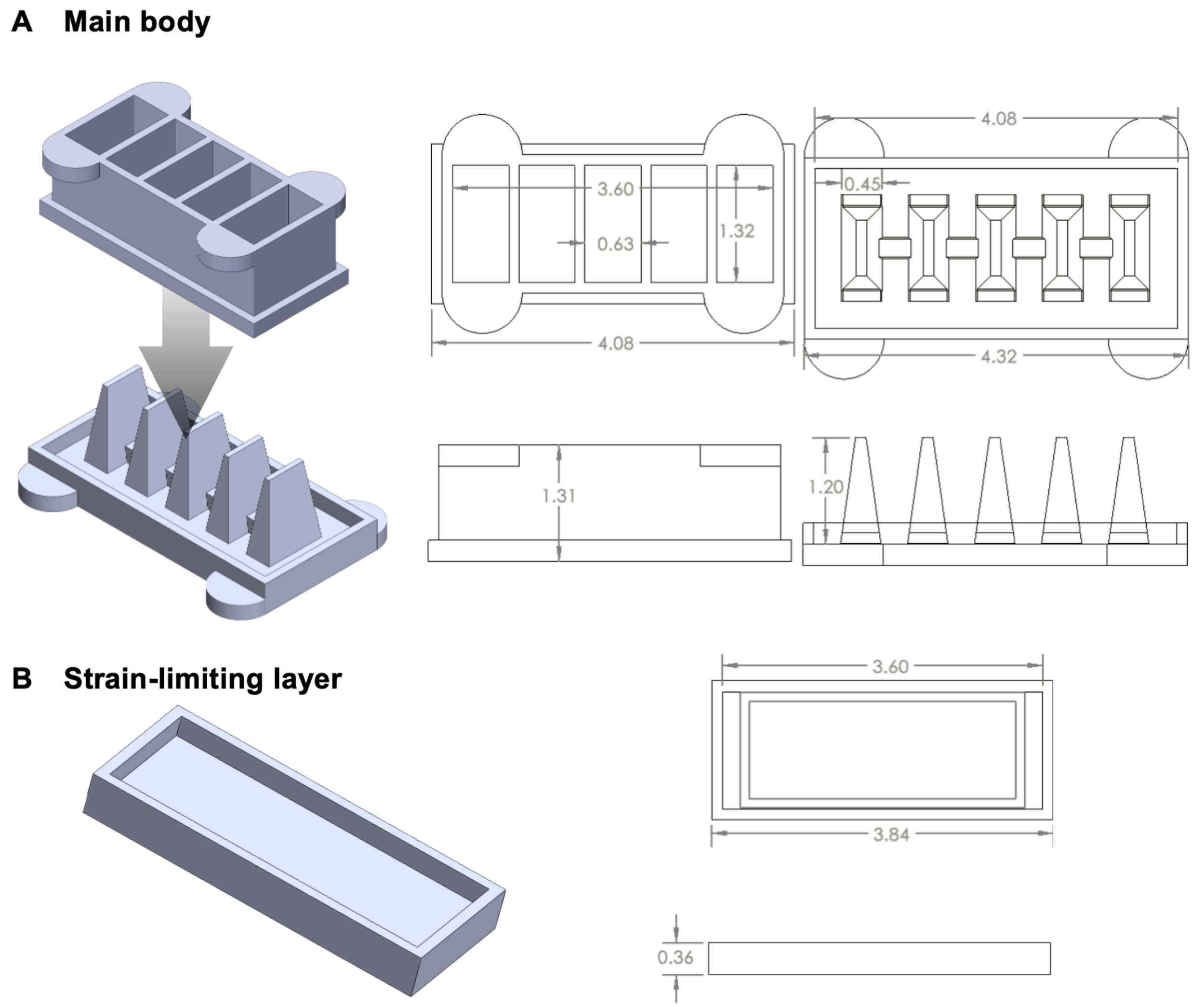

Fig. S10. Design and dimensions of pneu-net mold. (A) Mold assembly and dimensions in cm for the main body of the pneu-net, which is composed of the internal chambers and channels. (B) A rectangular base with a depth of 2 mm to create the strain-limiting layer. The pneu-net mold design was adapted from ref. *(61)*

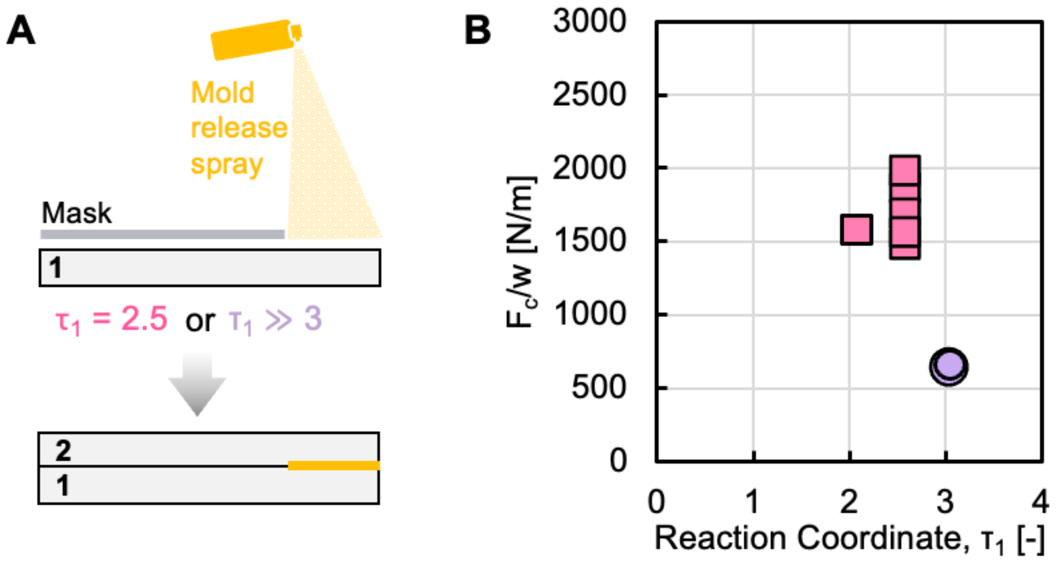

Fig. S11. Effect of mold release sprayed adjacent to adhesion bonding site. (A) Mold release spray was used to create the peel arms instead of non-stick transfer tape. (B) Results indicate that mold release agent away from the adhesion site does not affect the failure behavior (n = 8).

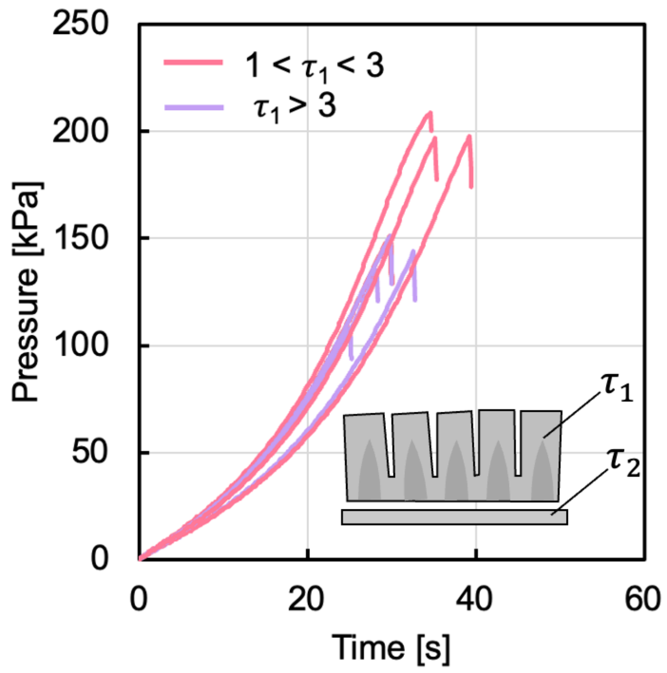

Fig. S12. Pressure as a function of time for pneu-net experiment. The pneu-net actuators were pressurized at a rate of 200 mL/min. The results show that the pneu-net that was bonded within the optimal bonding window ($1 < \tau_1 < \tau_1^*$) experienced bulk fracture at a higher pressure than the pneu-net that was bonded beyond the optimal bonding window (n = 8).

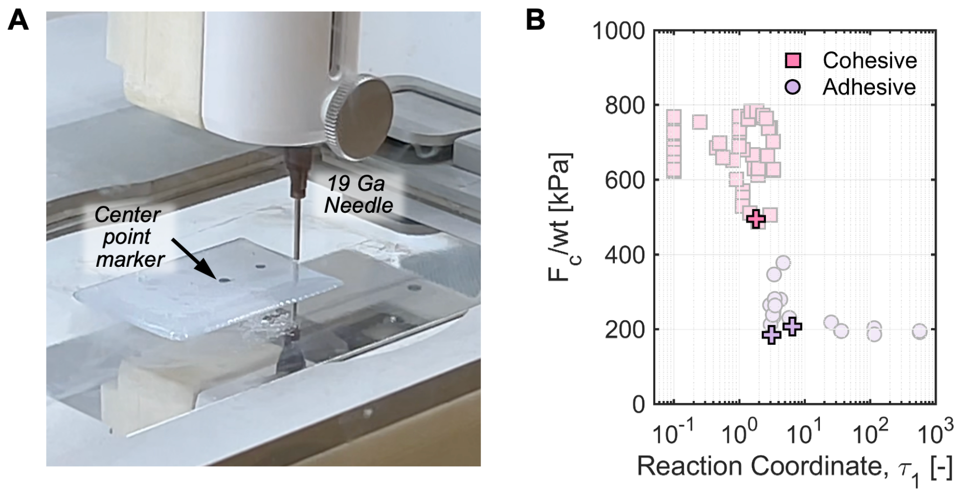

Fig. S13. T-peel test results from 3D printing Dragon Skin 30. (A) T-peel geometries were printed using Dragon Skin 30 with a print speed of 10 mm/s, 19 Ga needle, extrusion pressure of 200 kPa, and printbed temperature of 65 °C. The print parameters were chosen to accommodate the higher viscosity (approximately 7× that of Ecoflex 00-30). (B) T-peel results from the printed samples with varying τ_1 values (plus symbols), overlaid onto T-peel results for thickness of 2.5 mm at room temperature (Fig. 2B), indicate that the failure mode can be predicted even for direct ink writing of these platinum-catalyzed silicone elastomers by tracking the reaction coordinate.

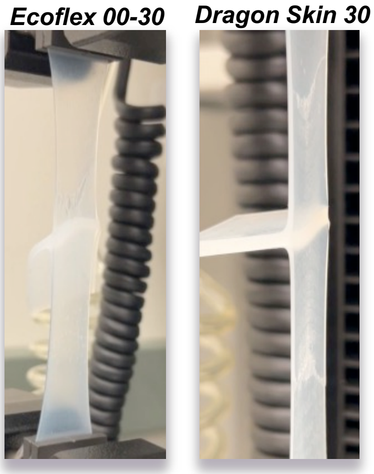

Fig. S14. T-peel samples fabricated via direct ink writing undergoing adhesive failure. The samples that were cured to a reaction coordinate greater than τ_1^* undergo adhesive failure. Strong in-plane adhesion is observed while the interface delaminates.

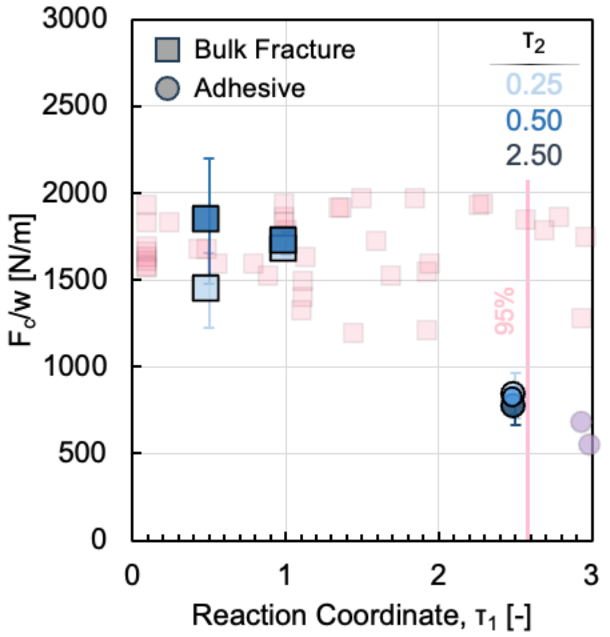

Fig. S15. Effect of the adhesive cure extent τ_2 on the failure behavior. T-peel test results from 7 combinations of τ_1 and τ_2 (blue). Each data point represents the average of 3 samples and the error bars are the standard deviations. Data from Fig. 2B is plotted in the background.

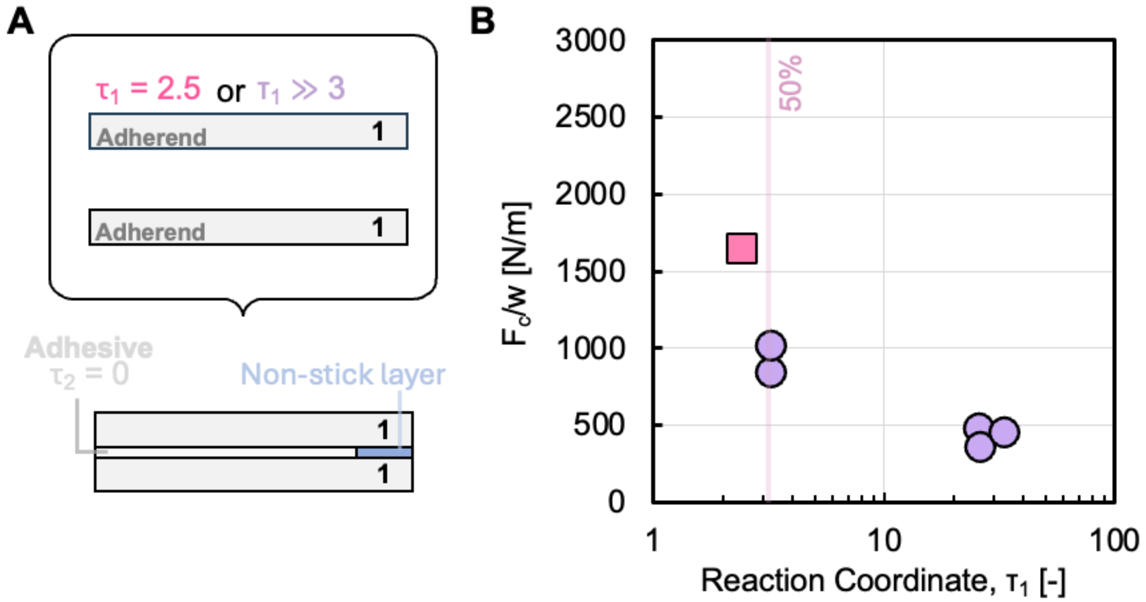

Fig. S16. Studying the failure behavior for multi-component assembly systems. (A) Two adherends were fabricated and assembled with the same τ_1 value by adhesion to each other via a layer of freshly mixed prepolymer ($\tau_2 = 0$). (B) The results indicate that we are able to predict the mode of failure solely based on the reaction coordinate of the adherend even in this multi-component assembly.

Table S1. E_a and ln(A) values for Ecoflex 00-30 and Dragon Skin 30.

Elastomer series	Frequency factor, ln(A [1/min])	Activation energy, E_a [kJ/mol]
Ecoflex 00-30	23.69	68.15
Dragon Skin 30	24.00	69.26

Table S2. τ_1 values for 95, 50, and 5% probability of bulk fracture for each material and thickness.

	Ecoflex 00-30			Dragon Skin 30
	**1.5 mm**	**2.5 mm**	**4.0 mm**	**2.5 mm**
95%	3.38	0.63	0.71	2.58
50%	3.52	0.82	0.80	3.13
5%	3.65	1.00	0.90	3.69

Movie S1. Pneumatic network actuators fabricated at different τ_1 values. The main body and strain-limiting base were assembled either within or beyond the optimal bonding window described in main text Fig. 6 showcase the importance of accounting for the reaction coordinate or extent of cure during a mold-based fabrication process.

Movie S2. Direct ink writing of platinum-catalyzed silicone elastomers. The T-peel geometry was 3D printed by extruding Ecoflex 00-30 on a heated printbed. By varying the time between printing two layers—in this case, acting as the adherend and the adhesive layer—we can tune the extent of cure to highlight the capabilities of the model to predict the different modes of failure for 3D printed elastomeric components purely based on the reaction coordinate.

REFERENCES AND NOTES

1. K. E. Polmanteer, Current perspectives on silicone rubber technology. *Rubber Chem. Technol.* **54**, 1051–1080 (1981).

2. P. Shivapooja, Q. Wang, L. M. Szott, B. Orihuela, D. Rittschof, X. Zhao, G. P. López, Dynamic surface deformation of silicone elastomers for management of marine biofouling: Laboratory and field studies using pneumatic actuation. *Biofouling* **31**, 265–274 (2015).

3. G. Wang, A. Li, W. Zhao, Z. Xu, Y. Ma, F. Zhang, Y. Zhang, J. Zhou, Q. He, A review on fabrication methods and research progress of superhydrophobic silicone rubber materials. *Adv. Mater. Interfaces* **8**, 2001460 (2021).

4. D. M. J. Dykstra, C. Lenting, A. Masurier, C. Coulais, Buckling metamaterials for extreme vibration damping. *Adv. Mater.* **35**, e2301747 (2023).

5. N. Rodriguez, S. Ruelas, J.-B. Forien, N. Dudukovic, J. DeOtte, J. Rodriguez, B. Moran, J. P. Lewicki, E. B. Duoss, J. S. Oakdale, 3D printing of high viscosity reinforced silicone elastomers. *Polymers* **13**, 2239 (2021).

6. A. Colas, J. Curtis, "Application of materials in medicine, biology, and artifical organs" in *Biomaterials Science: An Introduction to Materials in Medicine* (Elsevier, ed. 2, 2004), pp. 697–704.

7. N. MacCallum, C. Howell, P. Kim, D. Sun, R. Friedlander, J. Ranisau, O. Ahanotu, J. J. Lin, A. Vena, B. Hatton, T.-S. Wong, J. Aizenberg, Liquid-infused silicone as a biofouling-free medical material. *ACS Biomater Sci. Eng.* **1**, 43–51 (2015).

8. H. Yuk, T. Zhang, G. A. Parada, X. Liu, X. Zhao, Skin-inspired hydrogel–elastomer hybrids with robust interfaces and functional microstructures. *Nat. Commun.* **7**, 12028 (2016).

9. M. Singh, D. L. Teodorescu, M. Rowlett, S. X. Wang, M. Balcells, C. Park, B. Bernardo, S. McGarel, C. Reeves, M. R. Mehra, X. Zhao, H. Yuk, E. T. Roche, A tunable soft silicone bioadhesive for secure anchoring of diverse medical devices to wet biological tissue. *Adv. Mater.* **36**, e2307288 (2024).

10. K. R. Jinkins, S. Li, H. Arafa, H. Jeong, Y. J. Lee, C. Wu, E. Campisi, X. Ni, D. Cho, Y. Huang, J. A. Rogers, Thermally switchable, crystallizable oil and silicone composite adhesives for skin-interfaced wearable devices. *Sci. Adv.* **8**, eabo0537 (2022).

11. H.-C. Liu, Y. Zeng, C. Gong, X. Chen, P. Kijanka, J. Zhang, Y. Genyk, H. Tchelepi, C. Wang, Q. Zhou, X. Zhao, Wearable bioadhesive ultrasound shear wave elastography. *Sci. Adv.* **10**, eadk8426 (2024).

12. T. Suzuki, A. Kasuya, Adhesion of addition-reaction type silicone elastomers. *J. Adhes. Sci. Technol.* **3**, 463–473 (1989).

13. A. Borók, K. Laboda, A. Bonyár, PDMS bonding technologies for microfluidic applications: A review. *Biosensors* **11**, 292 (2021).

14. J. A. González Calderón, D. Contreras López, E. Pérez, J. Vallejo Montesinos, Polysiloxanes as polymer matrices in biomedical engineering: Their interesting properties as the reason for the use in medical sciences. *Polym. Bull.* **77**, 2749–2817 (2020).

15. B. Jumet, M. D. Bell, V. Sanchez, D. J. Preston, A data-driven review of soft robotics. *Adv. Intell. Syst.* **4**, 2100163 (2022).

16. A. Rajappan, B. Jumet, D. J. Preston, Pneumatic soft robots take a step toward autonomy. *Sci. Rob.* **6**, eabg6994 (2021).

17. F. Tauber, M. Desmulliez, O. Piccin, A. A. Stokes, Perspective for soft robotics: The field's past and future. *Bioinspir. Biomim.* **18**, 035001 (2023).

18. F. Ilievski, A. D. Mazzeo, R. F. Shepherd, X. Chen, G. M. Whitesides, Soft robotics for chemists. *Angew. Chem. Int. Ed. Engl.* **50**, 1890–1895 (2011).

19. T. J. K. Buchner, S. Rogler, S. Weirich, Y. Armati, B. G. Cangan, J. Ramos, S. T. Twiddy, D. M. Marini, A. Weber, D. Chen, G. Ellson, J. Jacob, W. Zengerle, D. Katalichenko, C. Keny, W. Matusik, R. K. Katzschmann, Vision-controlled jetting for composite systems and robots. *Nature* **623**, 522–530 (2023).

20. H. C. Lee, N. Elder, M. Leal, S. Stantial, E. Vergara Martinez, S. Jos, H. Cho, S. Russo, A fabrication strategy for millimeter-scale, self-sensing soft-rigid hybrid robots. *Nat. Commun.* **15**, 8456 (2024).

21. C. Arase, Q. Yu, K. Becker, "Bonding rigid and soft materials using flocking" in *2024 IEEE 7th International Conference on Soft Robotics (RoboSoft)* (IEEE, 2024), pp. 89–96.

22. D. Drotman, S. Jadhav, D. Sharp, C. Chan, M. T. Tolley, Electronics-free pneumatic circuits for controlling soft-legged robots. *Sci. Rob.* **6**, eaay2627 (2021).

23. D. J. Preston, P. Rothemund, H. J. Jiang, M. P. Nemitz, J. Rawson, Z. Suo, G. M. Whitesides, Digital logic for soft devices. *Proc. Natl. Acad. Sci. U.S.A.* **116**, 7750–7759 (2019).

24. B. Sparrman, C. Du Pasquier, C. Thomsen, S. Darbari, R. Rustom, J. Laucks, K. Shea, S. Tibbits, Printed silicone pneumatic actuators for soft robotics. *Addit. Manuf.* **40**, 101860 (2021).

25. M. Schaffner, J. A. Faber, L. Pianegonda, P. A. Rühs, F. Coulter, A. R. Studart, 3D printing of robotic soft actuators with programmable bioinspired architectures. *Nat. Commun.* **9**, 878 (2018).

26. Q. Yu, N. Cao, C. Folinus, K. P. Becker, Low-volume cores for fabrication of compact, versatile, and intelligent soft systems. *Adv. Funct. Mater.* **34**, 2404317 (2024).

27. V. Lampinen, A. Koivikko, M. Pihlajamäki, V. Sharma, V. Sariola, Soft pneumatic tactile pressure sensor matrix. *Rob. Rep.* **2**, 115–127 (2024).

28. O. D. Yirmibesoglu, J. Morrow, S. Walker, W. Gosrich, R. Canizares, H. Kim, U. Daalkhaijav, C. Fleming, C. Branyan, Y. Menguc, "Direct 3D printing of silicone elastomer soft robots and their performance comparison with molded counterparts" in *2018 IEEE International Conference on Soft Robotics (RoboSoft)* (IEEE, 2018), pp. 295–302.

29. S. Walker, E. Lingle, N. Troxler, T. Wallin, K. Healy, Y. Mengüç, J. R. Davidson, Predicting interfacial layer adhesion strength in 3D printable silicone. *Addit. Manuf.* **47**, 102320 (2021).

30. C.-U. Lee, K. C. H. Chin, A. J. Boydston, Additive manufacturing by heating at a patterned photothermal interface. *ACS Appl. Mater. Interfaces* **15**, 16072–16078 (2023).

31. D. W. Yee, A HAPPI solution: Photothermal additive manufacturing of unmodified thermoset resins. *Matter* **6**, 2599–2601 (2023).

32. C.-F. Chen, K. Wharton, Characterization and failure mode analyses of air plasma oxidized PDMS–PDMS bonding by peel testing. *RSC Adv.* **7**, 1286–1289 (2017).

33. J. Roth, V. Albrecht, M. Nitschke, C. Bellmann, F. Simon, S. Zschoche, S. Michel, C. Luhmann, K. Grundke, B. Voit, Surface functionalization of silicone rubber for permanent adhesion improvement. *Langmuir* **24**, 12603–12611 (2008).

34. M. A. Eddings, M. A. Johnson, B. K. Gale, Determining the optimal PDMS–PDMS bonding technique for microfluidic devices. *J. Micromech. Microeng.* **18**, 067001 (2008).

35. K. Jung, D. G. Kim, S. Jung, Adhesion of PDMS substrates assisted by plasma graft polymerization. *Surf. Interface Anal.* **48**, 597–600 (2016).

36. S. Park, K. Mondal, R. M. Treadway, V. Kumar, S. Ma, J. D. Holbery, M. D. Dickey, Silicones for stretchable and durable soft devices: Beyond Sylgard-184. *ACS Appl. Mater. Interfaces* **10**, 11261–11268 (2018).

37. K. P. Becker, Y. Chen, R. J. Wood, Mechanically programmable dip molding of high aspect ratio soft actuator arrays. *Adv. Funct. Mater.* **30**, 1908919 (2020).

38. T. J. Jones, E. Jambon-Puillet, J. Marthelot, P.-T. Brun, Bubble casting soft robotics. *Nature* **599**, 229–233 (2021).

39. A. Silva, D. Fonseca, D. M. Neto, M. Babcinschi, P. Neto, Integrated design and fabrication of pneumatic soft robot actuators in a single casting step. *Cyborg Bionic Syst.* **5**, 0137 (2024).

40. T. Wienzek, A. Seibel, Elastomeric prepregs for soft robotics applications. *Adv. Eng. Mater.* **21**, 1801200 (2019).

41. S. Walker, U. Daalkhaijav, D. Thrush, C. Branyan, O. D. Yirmibesoglu, G. Olson, Y. Menguc, Zero-support 3D printing of thermoset silicone via simultaneous control of both reaction kinetics and transient rheology. *3D Print. Addit. Manuf.* **6**, 139–147 (2019).

42. K.-S. Kim, J. Kim, Elasto-plastic analysis of the peel test for thin film adhesion. *J. Eng. Mater. Technol.* **110**, 266–273 (1988).

43. J. L. Gardon, Peel adhesion. I., Some phenomenological aspects of the test. *J. Appl. Polym. Sci.* **7**, 625–641 (1963).

44. J. Li, A. D. Celiz, J. Yang, Q. Yang, I. Wamala, W. Whyte, B. R. Seo, N. V. Vasilyev, J. J. Vlassak, Z. Suo, D. J. Mooney, Tough adhesives for diverse wet surfaces. *Science* **357**, 378–381 (2017).

45. M. D. Bartlett, S. W. Case, A. J. Kinloch, D. A. Dillard, Peel tests for quantifying adhesion and toughness: A review. *Prog. Mater. Sci.* **137**, 101086 (2023).

46. B. R. Freedman, J. A. Cintron Cruz, P. Kwon, M. Lee, H. M. Jeffers, D. Kent, K. C. Wu, J. C. Weaver, D. J. Mooney, Instant tough adhesion of polymer networks. *Proc. Natl. Acad. Sci. U.S.A.* **121**, e2304643121 (2024).

47. P. Rothemund, A. Ainla, L. Belding, D. J. Preston, S. Kurihara, Z. Suo, G. M. Whitesides, A soft, bistable valve for autonomous control of soft actuators. *Sci. Rob.* **3**, eaar7986 (2018).

48. B. J. Cafferty, V. E. Campbell, P. Rothemund, D. J. Preston, A. Ainla, N. Fulleringer, A. C. Diaz, A. E. Fuentes, D. Sameoto, J. A. Lewis, G. M. Whitesides, Fabricating 3D structures by combining 2D printing and relaxation of strain. *Adv. Mater. Technol.* **4**, 1800299 (2019).

49. M. McCandless, A. Gerald, A. Carroll, H. Aihara, S. Russo, A soft robotic sleeve for safer colonoscopy procedures. *IEEE Rob. Autom. Lett.* **6**, 5292–5299 (2021).

50. T. F. Yap, A. Rajappan, M. D. Bell, R. M. Rasheed, C. J. Decker, D. J. Preston, Thermally accelerated curing of platinum-catalyzed elastomers. *Cell Rep. Phys. Sci.* **5**, 101849 (2024).

51. A. N. Gent, R. H. Tobias, Effect of interfacial bonding on the strength of adhesion of elastomers. III. Interlinking by molecular entanglements. *J. Polym. Sci. Polym. Phys. Ed.* **22**, 1483–1490 (1984).

52. R.-J. Chang, A. N. Gent, Effect of interfacial bonding on the strength of adhesion of elastomers. I. Self-adhesion. *J. Polym. Sci. Polym. Phys. Ed.* **19**, 1619–1633 (1981).

53. H. Varner, T. Cohen, Explaining the spread in measurement of PDMS elastic properties: Influence of test method and curing protocol. *Soft Matter* **20**, 9174–9183 (2024).

54. E. Porte, S. Eristoff, A. Agrawala, R. Kramer-Bottiglio, Characterization of temperature and humidity dependence in soft elastomer behavior. *Soft Rob.* **11**, 118–130 (2024).

55. K. Kendall, Thin-film peeling-the elastic term. *J. Phys. Appl. Phys.* **8**, 1449–1452 (1975).

56. K. Kendall, The adhesion and surface energy of elastic solids. *J. Phys. Appl. Phys.* **4**, 1186–1195 (1971).

57. A. A. Griffith, VI. The phenomena of rupture and flow in solids. *Philos. Trans. R. Soc. A Math. Phys. Eng. Sci.* **221**, 163–198 (1921).

58. A. N. Gent, P. Vondráček, Spontaneous adhesion of silicone rubber. *J. Appl. Polym. Sci.* **27**, 4357–4364 (1982).

59. M. A. Bell, K. P. Bekcer, R. J. Wood, Injection molding of soft robots. *Adv. Mater. Technol.* **7**, 2100605 (2022).

60. J. Yi, E. Keaney, J. Zhang, C. J. Hansen, W. Zukas, J. Mead, Improved adhesion in elastomeric laminates using elastomer blends. *Rubber Chem. Technol.* **95**, 465–478 (2022).

61. B. Mosadegh, P. Polygerinos, C. Keplinger, S. Wennstedt, R. F. Shepherd, U. Gupta, J. Shim, K. Bertoldi, C. J. Walsh, G. M. Whitesides, Pneumatic networks for soft robotics that actuate rapidly. *Adv. Funct. Mater.* **24**, 2163–2170 (2014).
